# Divergent Evolution of Carbonaceous Aerosols during Dispersal of East Asian Haze

**DOI:** 10.1038/s41598-017-10766-4

**Published:** 2017-09-05

**Authors:** Wenzheng Fang, August Andersson, Mei Zheng, Meehye Lee, Henry Holmstrand, Sang-Woo Kim, Ke Du, Örjan Gustafsson

**Affiliations:** 1 Department of Environmental Science and Analytical Chemistry (ACES) and Bolin Centre for Climate Research, Stockholm University, Stockholm, 10691 Sweden; 2 College of Environmental Sciences and Engineering, Peking University, Beijing, 100871 China; 3 Department of Earth and Environmental Sciences, Korea University, Seoul, 02841 South Korea; 4 School of Earth and Environmental Sciences, Seoul National University, Seoul, 08826 South Korea; 5 Department of Mechanical and Manufacturing Engineering, University of Calgary, Calgary, T2N 1N4 Canada

## Abstract

Wintertime East Asia is plagued by severe haze episodes, characterized by large contributions of carbonaceous aerosols. However, the sources and atmospheric transformations of these major components are poorly constrained, hindering development of efficient mitigation strategies and detailed modelling of effects. Here we present dual carbon isotope (δ^13^C and Δ^14^C) signatures for black carbon (BC), organic carbon (OC) and water-soluble organic carbon (WSOC) aerosols collected in urban (Beijing and BC for Shanghai) and regional receptors (e.g., Korea Climate Observatory at Gosan) during January 2014. Fossil sources (>50%) dominate BC at all sites with most stemming from coal combustion, except for Shanghai, where liquid fossil source is largest. During source-to-receptor transport, the δ^13^C fingerprint becomes enriched for WSOC but depleted for water-insoluble OC (WIOC). This reveals that the atmospheric processing of these two major pools are fundamentally different. The photochemical aging (e.g., photodissociation, photooxidation) during formation and transport can release CO_2_/CO or short-chain VOCs with lighter carbon, whereas the remaining WSOC becomes increasingly enriched in δ^13^C. On the other hand, several processes, e.g., secondary formation, rearrangement reaction in the particle phase, and photooxidation can influence WIOC. Taken together, this study highlights high fossil contributions for all carbonaceous aerosol sub-compartments in East Asia, and suggests different transformation pathways for different classes of carbonaceous aerosols.

## Introduction

**Figure 1:**
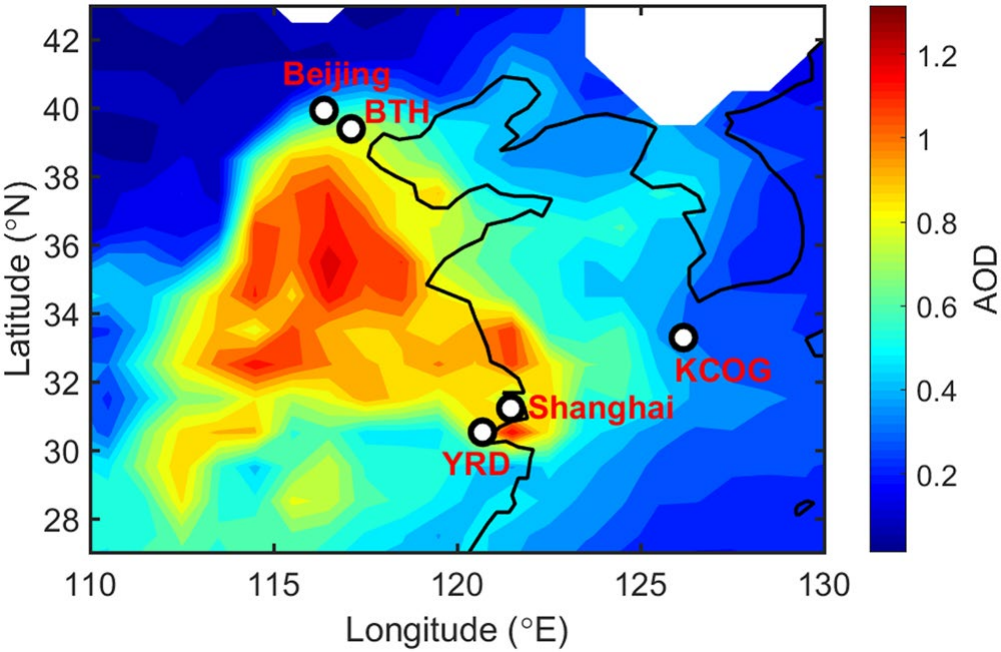
Sampling locations and monthly-averaged Aerosol Optical Depth (AOD) at 550 nm during the January 2014 multi-site campaign in East Asia. The black and white circles denote locations of sampling sites, including urban sites Beijing and Shanghai, and regional receptor sites BTH (Wuqing district at Tianjin, China), YRD (Haining, China), and KCOG (Korea Climate Observatory at Gosan, Jeju Island, South Korea). AOD data were obtained from NASA Moderate Resolution Imaging Spectroradiometer (MODIS) level 3 collection 6. The figure was created by MATLAB version R2015b (The MathWorks, Natick, MA, USA).

Thick smog shrouded Beijing and the northeast corridor of China in January of both 2013^1–4^ and 2014, spreading out over a vast area of East Asia (Fig. 1). These events propelled regional air pollution to the top of the agenda also for the public and policymakers. In some East Asian cities, the daily average PM_2.5_ (particulate matter with aerodynamic diameter ≤2.5 µm) levels for 2013 were more than ten times the World Health Organization (WHO) daily maximum level (25 µg m^−3^)^1, 5^. The Chinese Government has issued far-reaching policies to combat air pollution such as the ‘Action Plan for Air Pollution Prevention and Control (2013‒2017)’ and ‘Coordinated Development of Ecological Environment Protection Plan in Beijing-Tianjin-Hebei area’ (2015); with the latter plan including a mandatory 40% reduction in annual average concentrations of PM_2.5_ by 2020 in Beijing compared to 2013^6, 7^. However, achieving such ambitious goals remains a challenge in part due to large uncertainties concerning the contributions from different emission sources and complex atmospheric processes.

Carbonaceous aerosols (CA) – including black carbon (BC, or elemental carbon, EC) and organic carbon (OC) – are large components (35‒50%) of PM_2.5_^1^ in East Asia^8^. BC is the highly condensed carbonaceous residue derived exclusively from incomplete combustion of biomass and fossil fuel (e.g., coal and liquid petroleum)^9^. Conversely, OC is composed of water-soluble organic carbon (WSOC) and water-insoluble organic carbon (WIOC) and can either be emitted as primary particles from combustion and other primary sources or formed as secondary organic aerosol (SOA) via oxidation of natural and anthropogenic precursors.

Technology-based emission inventories (EI) estimate that anthropogenic BC and OC emissions from East Asia contribute more than 20% of current global emissions^8, 10, 11^. However, there exist large disagreements regarding the sources of ambient aerosols between source-diagnostic atmospheric observations and model-predicted source contributions (from technology-based emission inventory databases), implying potentially factors of 2–4 uncertainties in BC and OC EIs^10–12^. More specifically, this is reflected in the relative contributions from fossil fuel combustion (versus biomass burning) being systematically under-predicted by EI models relative to atmospheric-observational ^14^C-based diagnostic source apportionment of BC, for both South Asia and East Asia^3, 10, 13–16^.

Combustion-derived BC is the most important light-absorbing component of aerosols, exerting a positive, but highly uncertain radiative forcing (~0.2–1.0 W/m^2^)^9, 10, 17, 18^. The large uncertainty in assessing BC climate effects stems from uncertainties in emission estimates, effective light absorption and atmospheric lifetime^18^. In contrast, OC contributes mainly to cooling through both direct and indirect effects. The uncertainties regarding OC are perhaps even larger than for BC, given the chemical complexity of its dual primary and secondary formation pathways, and propensity for atmospheric transformations^19^. Studies have also shown that light-absorbing OC^19–21^ may contribute to positive aerosol forcing^22, 23^. A large fraction of OC is soluble in water, and thus classified as WSOC^2, 24–26^.

In recent years, studies based on dual-carbon isotopes (radiocarbon ^14^C/^12^C and stable carbon ^13^C/^12^C) have proven the ability to quantitatively constrain the relative contribution from different sources of carbonaceous aerosols^2, 3, 13–16, 25, 27^. The fossil sources are completely depleted in ^14^C while the biomass sources have a modern and constrained ^14^C/^12^C signature, allowing calculating the fraction of fossil versus biomass with high resolution. Moreover, the isotopic signatures of ^14^C/^12^C (∆^14^C) and ^13^C/^12^C (δ^13^C) can be combined in a 2D isotope signature with additional source-diagnostic and atmospheric processing information. For EC, the δ^13^C data for East Asia allows separation of fossil source into liquid fossil (e.g., traffic) and coal combustion, whereas the δ^13^C source signatures of OC pools are perturbed by atmospheric processing. So far, most ^14^C-based studies have focused on the total carbon (TC) or one specific CA isolate^1–3, 13–16, 27–29^. A simultaneous fingerprinting of the dual-carbon isotope composition of EC, OC, TC, WSOC, and WIOC in the same aerosol samples has the potential to advance our understanding of the sources of East Asian carbonaceous aerosols.

To this end, we here present δ^13^C/∆^14^C-diagnosed sources of EC, OC, WSOC, and WIOC in East Asia. Samples of the fine particles (PM_2.5_) were collected in January 2014 both at megacity source sites and rural receptor sites, including Beijing-Tianjin-Hebei (BTH, Beijing and Tianjin) area, Yangtze River Delta (YRD, Shanghai and Zhejiang), and Korea Climate Observatory at Gosan (KCOG, SE Yellow Sea, South Korea) (Supplementary Fig. S1). Markov-Chain Monte Carlo (MCMC) simulations were then employed to perform statistical source apportionment modelling of the two-dimensional isotopic signature of EC. The isotopic fingerprints of such near-synoptic aerosol samples provide integrated source constraints and additionally contribute constraints on atmospheric processing during long-range over-ocean transport of the East Asian haze. Knowledge about the sources and processing of carbonaceous aerosol components from this study facilitate an improved estimate of aerosol-induced climate and health impacts, and a scientific underpinning for developing a regionally-tailored mitigation strategy for different areas and source profiles in East Asia.

## Results and Discussion

### January 2014 regional aerosol regime

**Figure 2:**
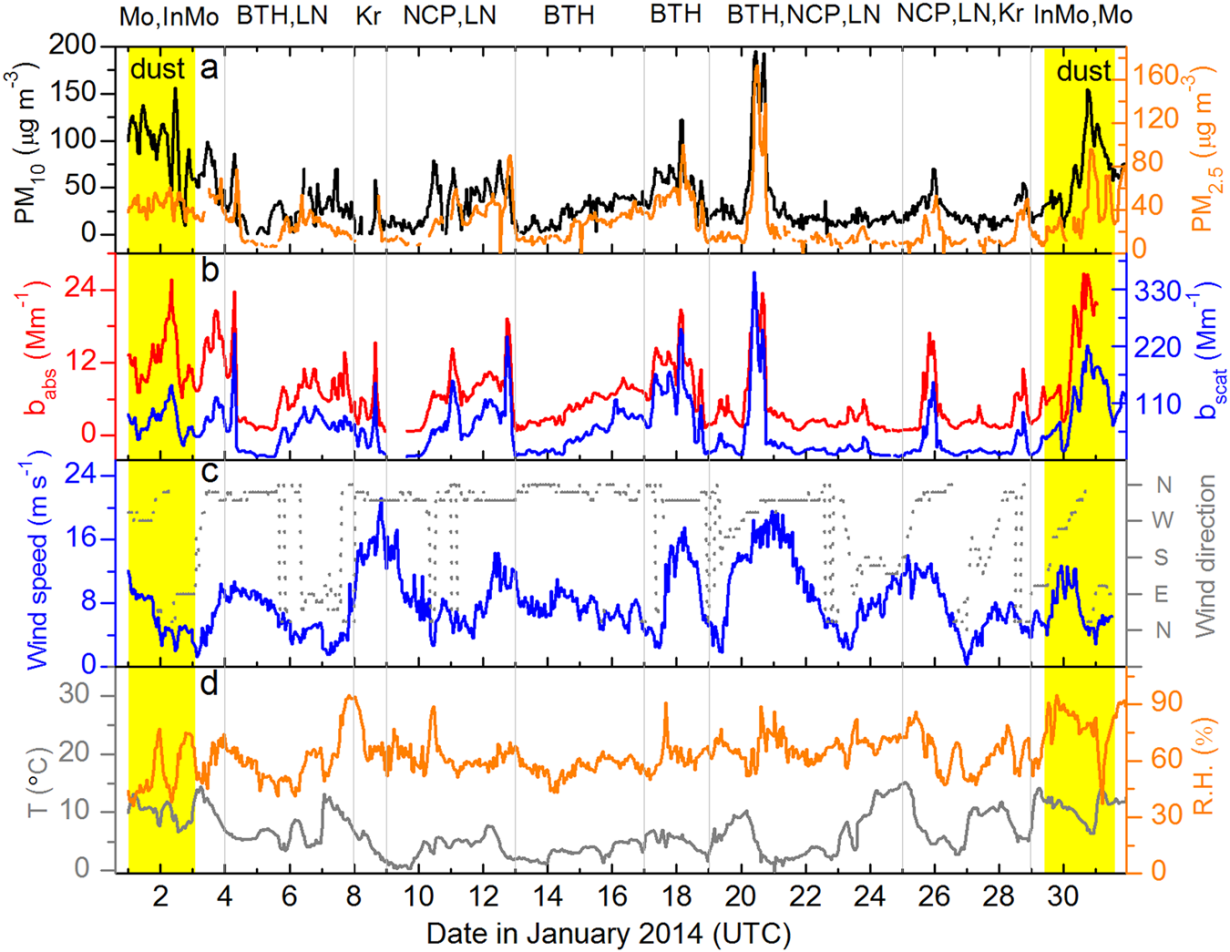
Time-resolved evolution of core aerosol properties and meteorological variables at KCOG in January 2014. (**a**) Variations of hourly-average mass concentrations of PM_2.5_ (right; orange, Bongseong (Jeju Island) and PM_10_ (left; black). (**b**) Absorption (*b*_abs_ at 528 nm, left; red) and scattering (*b*_scat_ at 550 nm, right; blue) coefficients with PM_1_ inlet, measured by Continuous Light Absorption Photometer (CLAP) and Nephelometer. (**c**) Wind speed (left; blue) and direction (right; grey) at KCOG. (**d**) Temperature (left; grey) and relative humidity (R.H., right; orange). Dominant back trajectory source clusters are marked on top of panels, and the dust events are marked in yellow. Mo, InMo, BTH, LN, Kr, and NCP refer to the source areas of Mongolia, Inner Mongolia, Beijing-Tianjin-Hebei, Liaoning province, Korea peninsula, and North China Plain, respectively.

High anthropogenic aerosol concentrations in January 2014 enveloped a vast area of East Asia (AOD > 0.3; Fig. 1), strongly affecting regional air quality and radiative budget. The analysis of NOAA HYSPLIT back trajectories (BT)^30^ indicate prevailing air mass transport from continental northeast Asia to the regional-scale receptor site KCOG during this time (Figs S2–12). Higher than average PM_2.5_ loadings (27.8 ± 21.6 µg m^3^) were observed at KCOG, and multiple air pollution episodes, where hourly concentration frequently reached 70 µg m^3^ with a peak of 173 µg m^3^ on 20 January, were recorded at the KCOG station (3‒4, 6‒8, 10‒12, 16‒20, 25‒26, and 28‒29 January) (Fig. 2). These pollution events were characterized by co-occurring high concentrations for multiple aerosol and gas species, for instance: aerosol light absorption (*b*_abs_ at 528 nm) and scattering (*b*_scat_ at 550 nm) frequently reached 20 and 250 Mm^−1^; several times higher than during background periods (Fig. 2). In addition, two obvious Asian dust storms, characterized by their elevated PM_10_/PM_2.5_ ratios, were observed during 1‒2 and 30‒31 January.

### Carbon aerosol concentrations at the urban and regional sites

**Figure 3:**
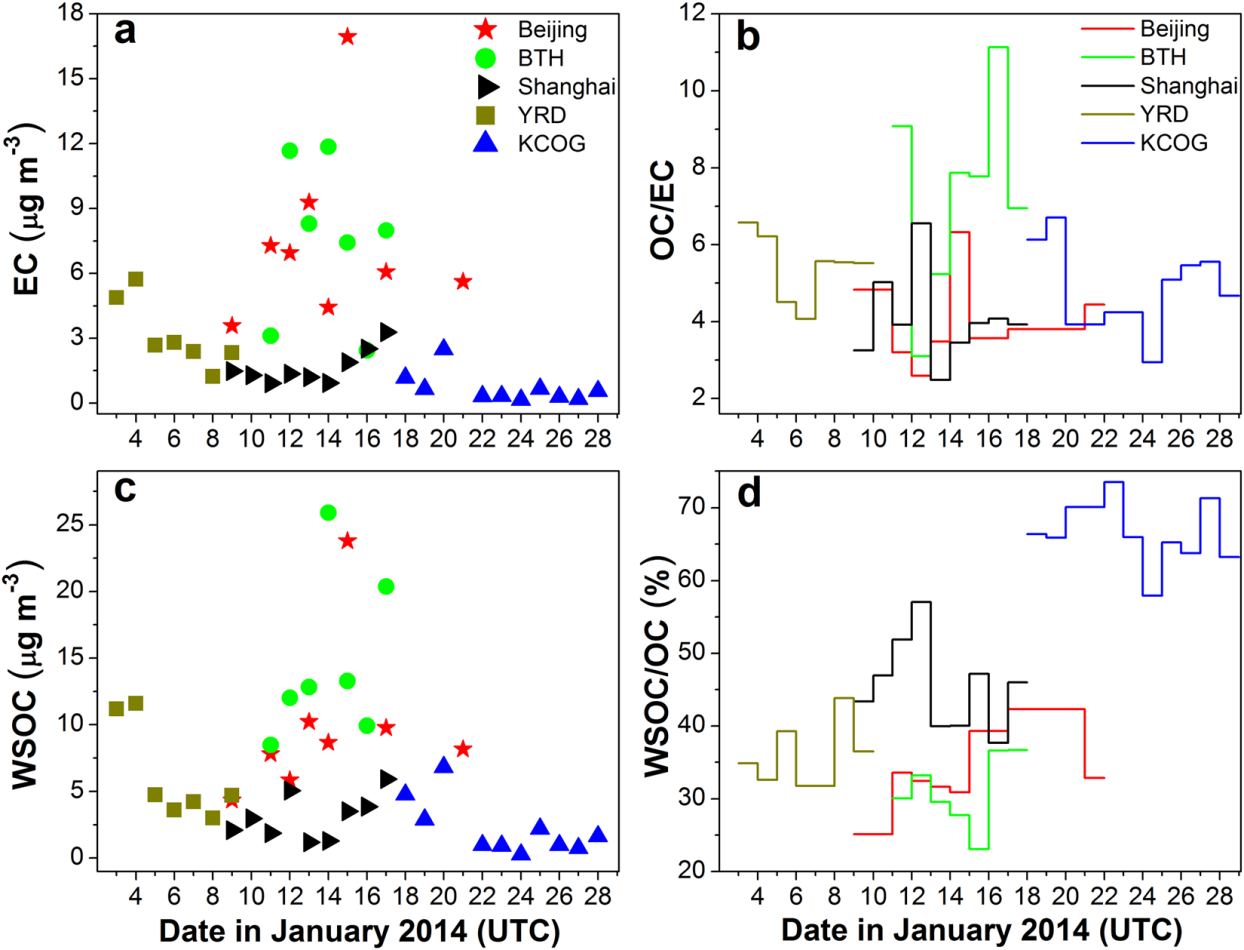
Temporal variations in concentrations of EC (**a**) and WSOC (**c**) as well as ratios of OC-to-EC (**b**) and WSOC-to-OC (**d**) in PM_2.5_ samples over East Asia in January 2014. Same color is employed for all the data from the same site. Symbols denote the concentrations and colored horizontal step lines indicate the ratios (OC/EC or WSOC/OC) at different sites. Data points shown on 18^th^ and 20^th^ January 2014 correspond to the sample ID of KCOG-0117 (17-18 January) and KCOG-0120 (20–21 January), respectively. The sampling durations for all the samples are given in Table S1.

Simultaneous measurements of EC, OC, WSOC, and WIOC (=OC − WSOC) concentrations at urban and rural receptor sites allow to investigate the synoptic atmospheric evolution of these carbon aerosol fractions. During January 2014, the PM_2.5_, EC, and OC concentrations were significantly higher at the sites in north China (Beijing, EC = 7.5 ± 3.9 μg m^−3^, OC = 28.4 ± 13.0 μg m^−3^; BTH, PM_2.5_ (117–353 μg m^−3^), EC = 7.5 ± 3.4 μg m^−3^, OC = 48.8 ± 21.3 μg m^−3^) compared to east China (Shanghai, EC = 1.7 ± 0.7 μg m^−3^, OC = 6.7 ± 3.3 μg m^−3^; YRD, PM_2.5_ (40–189 μg m^−3^), EC = 3.2 ± 1.5 μg m^−3^, OC = 18.2 ± 10.8 μg m^−3^), which in turn were higher than at the KCOG receptor site (EC = 0.7 ± 0.7 μg m^−3^, OC = 3.3 ± 2.9 μg m^−3^) (Fig. 3 and Table S1). Taken together, this shows that the air pollution was most pronounced in the northern region, but also that it indeed is a phenomenon extending over a large region, since the levels of carbon aerosol fractions at the inland regional sites (BTH and YRD) were on the same scale as their urban/megacity counterparts (Beijing and Shanghai) (Figs 3 and S13, and Table S1).

The January 2014 regional-scale haze in China is unfortunately not an isolated event; similar characteristics were also found for, e.g., January 2013. Concentrations were similar or slightly higher in January 2014 (Fig. S13 and Table S1) compared with the concentration measurements in January 2013 for BTH (EC: 3.5 ± 2.1 μg m^−3^, OC: 32.9 ± 16.8 μg m^−3^)^3^, YRD (EC: 2.3 ± 0.9 μg m^−3^, OC: 17.7 ± 5.3 μg m^−3^)^3^, and Beijing (EC: 5.58 ± 1.4 μg m^−3^, OC: 32.3 ± 22.6 μg m^−3^)^31^. For this more comprehensive January 2014 investigation, it was found that there is a significant inter- and intra-site variability of the OC/EC-ratio (~2‒11), suggesting partly decoupled emissions sources and influence by atmospheric processing (Fig. 3b). Some high OC/EC-ratio (e.g., 16 January at BTH) is probably due to large secondary organic aerosol formation.

The observed mean WSOC concentrations in PM_2.5_ for Beijing (9.8 ± 5.6 μg m^−3^), BTH (14.7 ± 5.8 μg m^−3^), Shanghai (3.1 ± 1.6 μg m^−3^), YRD (6.2 ± 3.4 μg m^−3^), and KCOG (2.2 ± 2.0 μg m^−3^) spanned ranges of 4.3–23.8, 8.5–25.9, 1.2–5.9, 3.0–11.6, and 0.3–6.8 μg m^−3^, respectively (Fig. 3c, Table S1). Similar WSOC concentrations in Beijing was reported for January 2013 (10.8 ± 3.1 μg m^−3^)^31^. The contributions of WSOC to OC were ~40% for the mainland China sites, but increased significantly during long-range transport to KCOG (66%), suggesting a significant difference in OC regimes (Fig. 3d). The WSOC concentrations at the different sites during the 2014 winter campaign were in good agreement with previous findings in East Asia^2, 26, 31–33^, while elevated WSOC levels and WSOC/OC ratios were here observed at KCOG.

### ∆^14^C -based source apportionment

**Figure 4:**
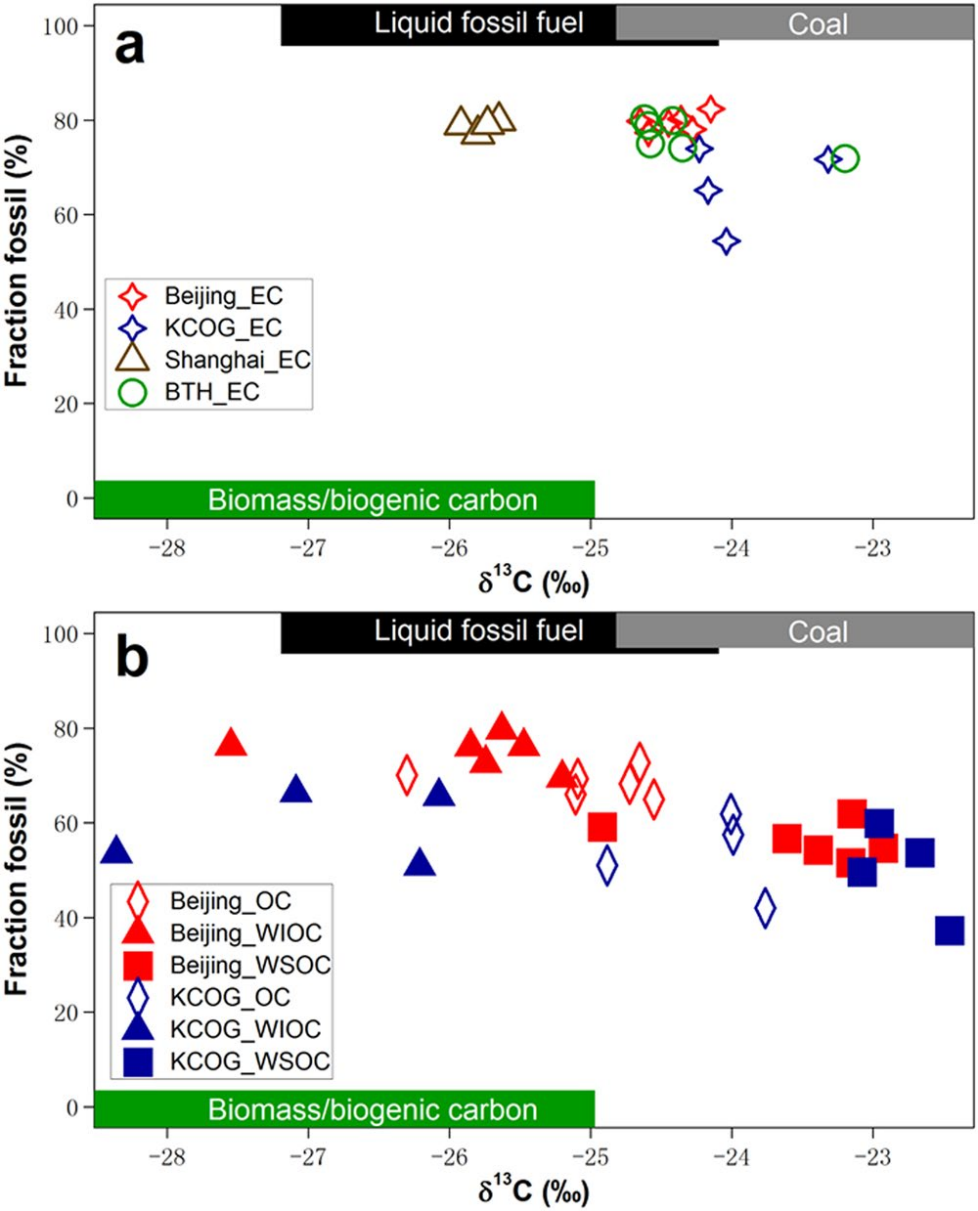
Two-dimensional carbon isotope ∆^14^C versus δ^13^C source apportionment of EC (**a**), OC, WSOC, and WIOC (**b**) for Beijing, BTH, Shanghai, and KCOG. The *f*_fossil_ of EC, OC, WSOC, and WIOC are calculated from observed ∆^14^C data and end-member constraints by equation (1). The δ^13^C source end-member ranges (mean ± sd) for biomass, liquid fossil, and coal are outlined by shaded rectangles within the ∆^14^C-based end-member ranges for biomass combustion or biogenic source (green, bottom), liquid fossil (black, top), and coal (grey, top).

Samples were selected for carbon isotope analysis from these high-pollution episodes from both source regions and the receptor stations (see Fig. S13 and Table S2). The synoptic weather patterns (Figs S14–19) and BTs indicate that the KCOG samples intercepted the main prevailing outflows from BTH and northern China. Here we find that the natural abundance radiocarbon (∆^14^C) signatures showed large fractional contributions from fossil fuels (fraction fossil, *f*_fossil_ > 50%) for all carbon fractions (EC; OC; WSOC; WIOC) in the January 2014 East Asian haze for all sites, suggesting that the aerosol regime in general is dominated by fossil combustion sources regardless of the division between primary emission or secondary formation (Fig. 4, Table S2). The EC, from primary incomplete combustion emissions, is characterized by high fossil contributions over both mainland China (Beijing 79 ± 2%; BTH 77 ± 3%; Shanghai 79 ± 1%) and in the SE Yellow Sea receptor region represented by KCOG (~66 ± 9%) (Fig. 4a). These values of *f*_fossil_ of EC are comparable to those reported for the few previous studies for this region, but more fossil than what is found for South Asia where a 50/50 balance is more common^3, 13–15, 28, 34^.

The OC, on the other hand, is formed from both incomplete combustion and other emission sources. Both primary and secondary OC are often considered to stem mainly from biomass burning/biogenic sources. However, the fossil contribution to WSOC in Beijing and KCOG in the present study are significantly higher compared to what had been found for WSOC in previous reports for South Asia (8–22%)^25^, (14–29%)^35^, Europe (4–24%)^36^, and North America (19–33%)^37^, (0–25%)^38^. Here, we add to recent literature to show that OC is even more dominantly fossil in wintertime Beijing^39^ and KCOG^2^. Intriguingly, the two main components of OC, WSOC and WIOC, display clear source differences: WIOC is more fossil at both Beijing (75 ± 3%) and KCOG (59 ± 3%) compared to WSOC (Beijing 56 ± 3%; KCOG 50 ± 8%) (Fig. 4b). Additionally, we note that all three major carbonaceous aerosol pools (EC, WSOC, WIOC) are more fossil in Beijing than at KCOG. The slightly lower *f*_fossil_ of carbon-aerosol fractions at KCOG is likely a consequence of a slight mixture of multiple sources during the long-range air transport. However, *f*_fossil_ of each carbon component for synoptic aerosol samples from Beijing and KCOG differ by only 10–25%.

### Dual carbon-isotope based source apportionment of EC

EC is highly recalcitrant to oxidation in the atmosphere. The δ^13^C signature of EC is therefore not affected by atmospheric processing to any large extent^3, 27^. The δ^13^C signature of EC (as opposed to OC) is therefore an important marker for EC emissions sources. For the present campaign, the ∆^14^C signatures of EC for Beijing, BTH, and Shanghai sites are close to the same horizontal line, and as low as −800‰ (Fig. S20), indicating their similarly high *f*_fossil_ (Fig. 4a). In contrast, the δ^13^C signals for Shanghai-EC (~−25.8 ± 0.1‰) is more depleted relative to that of Beijing-EC (~−24.4 ± 0.2‰) and BTH-EC (~−24.3 ± 0.5‰) (Figs 4a and S21). The ∆^14^C for EC at KCOG (~−625‰) has a higher biomass contribution, yet the δ^13^C (~−23.9 ± 0.4‰) is also depleted compared to Shanghai and remains in the range of EC for N. China (Beijing and BTH) source region.

**Figure 5:**
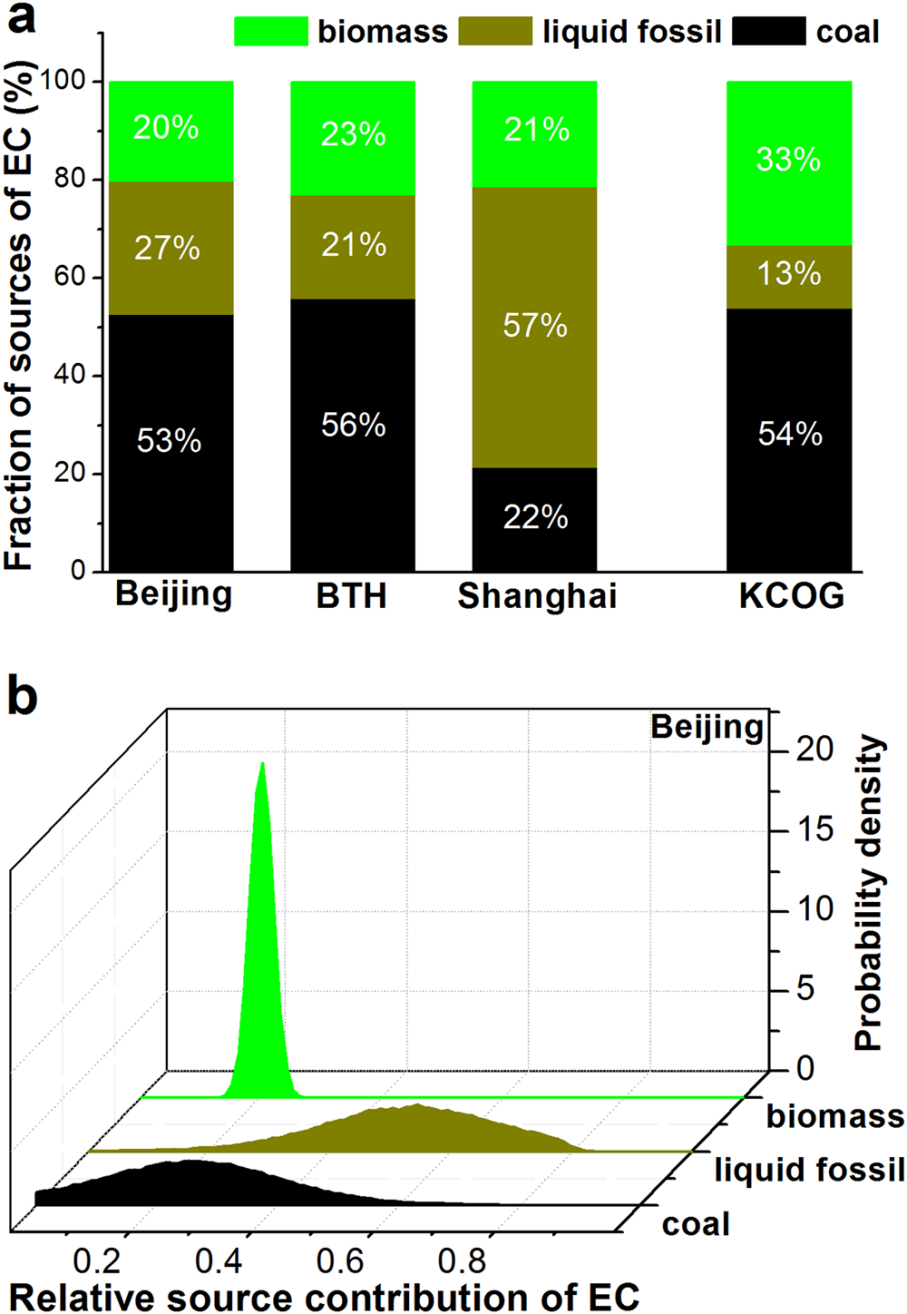
Fractions sourced from biomass, liquid fossil, and coal combustion to EC in Beijing, BTH, Shanghai, and KCOG (**a**) and posterior probability density function (PDF) of relative source contribution for EC illustrated for Beijing (**b**).

The three main emissions sources for EC in East Asia are: biomass burning, liquid fossil combustion (e.g., traffic) and coal combustion. These three broad categories can be differentiated also by means of their δ^13^C signatures. By combining the δ^13^C and ∆^14^C signatures, a means for resolving the relative contributions for these source categories for EC by isotopic mass balance is obtained. A Bayesian Markov chain Monte Carlo approach (MCMC) is used to account for the variability of the isotopic signatures from the different sources (see Table S3 for isotopic source signatures/endmembers)^3^. The results of the MCMC calculations are the posterior probability density functions (PDF) for the relative contribution of the three EC sources (Figs 5b and S22). To improve the precision of the estimated source fractions we combine several data points from each sampling site in the MCMC simulations^3^. Using the fraction coal as an example, the precision increases for combined data relative to for isolated data points (comparison illustrated in Fig. S23). For KOCG the variability in EC-*∆* ^14^C is larger than the expected endmember variability, suggesting variable source contributions and thereby challenging the MCMC averaging procedure implemented here. However, to allow comparisons of the overall source regimes affecting the different sites, this method is also implemented for KCOG, although the direct interpretation of estimated uncertainties is more complicated compared to the other stations. The results for the biomass burning contribution is similar to that acquired from ∆^14^C directly (Tables S2 and S4). The additional information provided by the MCMC of combined δ^13^C and ∆^14^C data is the further refinement and deconvolution of the fossil fraction into the two sub categories coal (*f*_coal_) and liquid fossil fuel (*f*_liq fossil_). The MCMC results reveal that the contributions of coal combustion were dominant for Beijing-EC (53 ± 14%), BTH-EC (56 ± 13%), and KCOG-EC (54 ± 11%) relative to that for Shanghai-EC (22 ± 14%) (Fig. 5a, Table S4). The larger proportion coal combustion for Beijing-EC and BTH-EC can be connected to the extensive coal use for residential heating in northern China during the heating season^40^, in addition to source contributions from coal-fired sectors such as the industry and power plant. Recent Weather Research and Forecasting model with Chemistry (WRF-Chem) simulations also indicate that residential solid fuel (coal and biomass) burning contributes far more to primary PM_2.5_ emissions in Beijing and the surrounding region than the transportation and power sectors combined, and more than all industry in winter^41^. The relatively lower contributions from liquid fossil fuel (e.g., transport) and biomass burning compared to coal combustion still emitted notable EC, since the total EC burden in Beijing and BTH region were extraordinary large. Contrary to Beijing-EC, Shanghai-EC was comparably less affected by coal burning, while it was more affected by liquid fossil sources. Implementation of a more restricted vehicle emission control strategy for Shanghai and surrounding area may thus be an effective way to reduce not only EC aerosols but also OC aerosols for this region.

The structure of fuel/source contributions to EC shown in Fig. 5a depicts a clear geographical source fingerprint: northeast China (Beijing and BTH) and KCOG share a similar mixture with coal combustion dominating EC, while East China (i.e., YRD) EC has a greater imprint from liquid fossil fuel combustion. These observationally-based constraints on EC sources may contribute to eventually improved radiative forcing assessments, as not only improved emission fluxes, but also combustion source types matter, as it has been found that fossil-fuel-dominated EC plumes are approximately 100% more efficient warming agents compared to biomass-burning-dominated plumes^42^. The similar source signatures of KCOG-EC, Beijing-EC, and BTH-EC adds to the synoptic and BT analysis, suggesting that the larger-footprint East Asia EC aerosol, as reflected by the KCOG, were prevailingly affected by air transport over northeast China (BTH) during the sampling period.

### Divergent atmospheric processing of WSOC and WIOC

The δ^13^C signature of carbonaceous aerosols is affected by both sources and atmospheric processing. In contrast to EC, OC is less recalcitrant to atmospheric processing and the δ^13^C for OC may thus be modulated by kinetic isotope effects (KIE) of atmospheric reactions during aerosol formation and atmospheric aging^2, 25, 35, 43–45^. While there is a deficit of studies on the δ^13^C shifts due to atmospheric reactions, the available data suggest that gas-phase products formed from oxidations of VOCs are depleted in δ^13^C signals compared to their precursors^46^, which is consistent with the expected direction for a KIE. One study on δ^13^C isotopic effects during SOA formation suggested that some SOA products were isotopically depleted, while the effect of other SOA products was sometimes so small that the δ^13^C of the particulate products remained inseparable to that of the precursor^47^. Laboratory studies of KIE have also found that particulate SOA formed from OH-initiated photooxidation of toluene (as representative of petroleum emissions) is depleted in δ^13^C^44^ while δ^13^C isotopic effect of biogenic β-pinene SOA was so small and indistinguishable from its precursor^48^. Taken together, these observations may suggest that the δ^13^C isotope effect during SOA formation indeed may induce total SOA products to be isotopically depleted. Since there are great diversity of emission precursors, SOA products, and SOA formation pathways^49–51^, the limited number of observations and large uncertainties of the isotopic effects on δ^13^C during SOA formation call on more measurements to constrain the δ^13^C effects during SOA formation, partitioning, and aging.

In contrast to the direction for δ^13^C shift due to SOA formation, observations have shown that photochemical aging of organic aerosols induces a fractionation in the aerosol δ^13^C, leading to more positive δ^13^C values in the remaining aerosols, consistent with KIE-induced preferential removal of lighter carbon isotopes (^12^C)^45^. These observations on δ^13^C shifts for secondary aerosol formation and for atmospheric photochemical aging, taken together, have led to the working hypothesis that the direction of δ^13^C evolution from source regions to receptor regions may be employed to indicate the relative dominance of fresh SOA formation vs aerosol photochemical aging during long-range transport^2, 35^.

**Figure 6:**
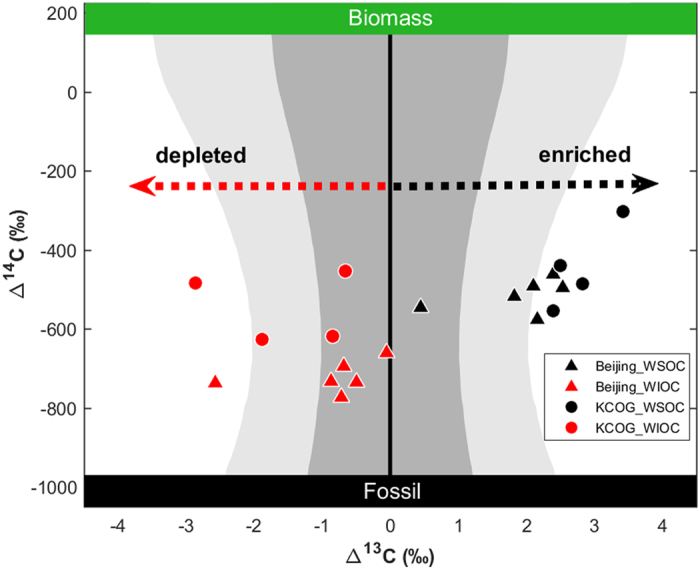
Comparison of ∆^13^C (=δ^13^C_observed_ − δ^13^C_source-predicted_) versus ∆^14^C of WSOC and WIOC at Beijing and KCOG. ∆^13^C denotes the difference in δ^13^C between δ^13^C signals of observed and source mixture predicted by MCMC simulations for each WSOC (or WIOC) sample (that is, ∆^13^C = δ^13^C_observed_ − δ^13^C_source-predicted_). The dual-isotope signatures of the original sources are marked by grey shade (dark grey and light grey represent 1σ and 2σ numerical spread, respectively), including coal, fossil fuel, and biomass/biogenic sources. The δ^13^C source endmember databases are found in Table S3.

The present study offers the opportunity to explore the δ^13^C-diagnosed CA dynamics between source and receptor regions in East Asia with the sampling in the same winter haze period, and also for different CA components. We first note that WSOC is more enriched in δ^13^C relative to OC, WIOC, TC, and EC, for both source-region Beijing and receptor-region KCOG (Figs 4 and S21). To compare the effects of atmospheric processing during long-range transport on both the WSOC and the WIOC pools, the ∆^13^C (=δ^13^C_observed_ − δ^13^C_source-predicted_) signatures were computed. The ∆^13^C is a means for decoupling the effects of atmospheric processing from the expected source signatures, details about the computation are given in the Methods. By combining ∆^14^C and ∆^13^C, we can isolate sources and atmospheric processes in the two dimensions (Fig. 6).

The ∆^13^C results show that both WSOC and WIOC are strongly influenced by atmospheric processing, but in opposite directions (Fig. 6). For WSOC, there is an enrichment in δ^13^C relative to predicted source values for all observations in both Beijing region and especially at the distant receptor KCOG, where there is several per mil of induced net fractionation. The difference in δ^13^C signatures for each carbon fraction between source and receptor provide a direct comparison of the effects of atmospheric processing for the different carbon components. The δ^13^C of recalcitrant EC show very little difference between source (Beijing and BTH) and receptor (KCOG), suggesting no (or very limited) atmospheric processing of this recalcitrant CA fraction (Fig. 4a). In contrast, all the δ^13^C signals of KCOG-WSOC (−22.8 ± 0.2‰) are well outside the initial source range and are more enriched compared to Beijing-OC (−25.1 ± 0.6‰), and Beijing-WSOC (−23.5 ± 0.7‰), indicating that WSOC is more likely affected by atmospheric oxidation/ageing during atmospheric transport compared to not only EC but also relative to other OC components (Figs 4b and S20–21). The observed enrichment in δ^13^C of WSOC is consistent with simultaneously increased WSOC/OC ratios during the source-receptor transport, suggesting a sizeable effect of photochemical aging on OC pools (Fig. 3d). During atmospheric oxidation, some hydrocarbons are likely transformed into multifunctional OC with higher oxidation state and polarity, increasing their solubility in water^43, 52^. This photochemical aging transforms more hydrophobic OC aerosols to become more hydrophilic OC. The formed WSOC (e.g., carboxylic acids) can break down to release CO_2_/CO or short-chain VOCs during transport, whose isotopic composition would be δ^13^C-depleted because of the KIE effect, whereas the remaining aerosol WSOC becomes increasingly enriched in δ^13^C^45, 53^. In addition, the high relative humidity in the air during the oversea transport may facilitate the WSOC partitioning in the particle phase.

In contrast to WSOC, fossil-dominated WIOC in Beijing clusters around the δ^13^C of the source endmember range, yet with all observations on the depleted side (Fig. 6). After long-range oversea transport to KCOG, the ∆^13^C_WIOC_ signals are all negative (depleted). An explanation for the lower extent of isotope fractionation for WIOC compared to WSOC is that many fossil components in WIOC are relatively more recalcitrant than WSOC^54^. The lower *f*_fossil_ of WSOC may reflect that OC of biomass burning origins are more water soluble^55^ and that oxidative aging of carbonaceous aerosols hence is preferential for more ^14^C-modern (i.e., biomass) components^2, 25, 35^. It is striking that the shift in ∆^13^C of WIOC and WSOC are in diametrically opposed directions, implying fundamentally different atmospheric processing and/or emission ratios of these two major components of carbonaceous aerosols in the East Asian haze.

In addition to a depletion of ∆^13^C, suggesting formation of WIOC through secondary processes, aging may also occur through rearrangement reactions (e.g., oligomerization/polymerization) and other transformations of some of the WIOC pool into the WSOC pool^38^, generating less hygroscopic large molecules within the particle phase^56, 57^. Since there is no net transfer of total carbon, these reactions do not affect δ^13^C signature of the total carbon (TC). However, these processes may redistribute WSOC and WIOC pools and shift the δ^13^C signature of a subpopulation, and can contribute to depleted signature in δ^13^C of WIOC. Clearly, the WSOC and WIOC compartments are resulting from diametrically different sources, formation, and aging.

### Implications

A vast area of East Asia experience severe and persistent aerosol pollution during winter. Our quantitative isotope-based constraints reveal that fossil sources are dominant for not only EC, but also for WIOC and WSOC aerosols in East Asia winter, yet with EC showing the highest relative fossil contribution. Emissions from fossil fuel combustion are a key target to combat the carbonaceous aerosol pollution. The δ^13^C/∆^14^C-based MCMC calculations of EC aerosols show that the contribution of coal combustion in Beijing-Tianjin-Hebei region are larger than liquid fossil and biomass burning combined, highlighting that reducing coal combustion and improving its burning efficiency should be top priority for efforts to fight EC and also (co-emitted) OC in this area. Reducing EC emissions by reduction and replacement of high-emitting end-use coal-fired combustion processes with cleaner energy across various sectors or by using cleaner residential stoves for space heating and cooking instead of ones with uncontrolled and inefficient combustion of coal will limit emissions, benefiting both climate and air quality. In parallel, controlling emissions from liquid-fossil-fueled vehicles should be another priority in Beijing and especially Shanghai megacities. Eliminating emissions from biomass burning (e.g., heating, open fires of crop residue) could be an efficient strategy in all regions.

In general, the isotopic fingerprints of synoptic samples in East Asia provide temporal and geographic source constraints and allow assessing the atmospheric processing that is occurring during over-ocean long-range transport. In forthcoming modelling efforts, the OC aerosols should be treated differently for WSOC and WIOC sub-compartments, to reflect their divergent evolution and different atmospheric processing. This top-down observation-based quantification of the sources of the different carbon aerosol components provides improved input for the aerosol parameterization in atmospheric chemistry transport models and to test/improve existing ‘bottom-up’ technology-based emission inventories of BC, OC, WIOC, and WSOC for East Asia, as well as a credible scientific underpinning for efficient mitigation actions toward efficiently reducing anthropogenic aerosol emissions.

## Methods

### The East Asia sampling campaign

The intensive wintertime aerosol pollution campaign was conducted at Beijing, Wuqing (BTH), Shanghai, Haining (YRD), and Korea Climate Observatory at Gosan (KCOG) during January 2014. Detailed description of sampling sites, sampling duration for each site, and instrumentation are provided in Supplementary Fig. S1, Tables S1 and S5, and Text S1. Daily aerosol samples were collected on pre-combusted quartz-fiber filters (Millipore) using high volume PM_2.5_ samplers^2, 3, 31^. At least three filter blanks were collected for each site throughout the duration of the campaign. The filter samples collected at the five sites were analyzed for concentrations of OC, EC, TC, and WSOC. The measurements of PM_2.5_ and PM_10_ (KCOG and Bongseong on Jeju Island) mass concentrations, aerosol absorption (*b*_abs_) and scattering (*b*_scat_) coefficients with PM_1_ inlet and meteorological data were collected at regional receptor site KCOG with high frequency (averaged to ≤1 h, Fig. 2). *b*_abs_ and *b*_scat_ were continuously measured by Continuous Light Absorption Photometer (CLAP) and Nephelometer^58, 59^.

### Concentration measurements of carbonaceous aerosols

The carbonaceous aerosol concentrations (EC and OC) were measured by a thermal-optical transmission (TOT) analyzer (Sunset Laboratory Inc., Tigard, OR, USA) using the National Institute for Occupational Safety and Health (NIOSH) 5040 method^60^. The aerosol samples were acidified prior to analysis, by fumigation in open glass Petri dishes kept in a desiccator over 12 M hydrochloric acid for 24 h and subsequently dried at 60 °C for 1h, to remove the carbonates^2, 25, 35^. TC represents OC and EC, assuming inorganic carbon is equal to carbonates. The comparison of OC/EC concentration measurements between the acid fumigation of filter punches and non-acid treatments showed only small to no differences of less than 5% (e.g., Table S6 show the comparison for Shanghai samples). Thus, this treatment was unlikely to affect the overall carbon fraction concentration and further isotope measurements of EC and TC.

The analytical protocol for WSOC concentration measurement was described previously^2, 25, 35^. Briefly, WSOC was extracted by ultrasonication, centrifugation, and filtration of the supernatant using 0.02-μm cutoff aluminum syringe filters (Anotop 10 Plus; Whatman, Kent, UK) and quantified by a high-temperature catalytic oxidation instrument (Shimadzu-TOC-VCPH, Japan). TC and WSOC concentration values were blank corrected by subtracting an average of the field blanks. The mean relative standard deviation of triplicate analysis was <5% for EC, TC, and WSOC. No EC was detected in blanks. The instruments were frequently calibrated by standards and other reference materials.

### Carbon isotope analysis

Synoptic samples with high carbon loadings, corresponding to high-pollution events, were selected for isolation of the different carbon components and their isotope measurements. For EC and TC isolation, a filter area corresponding to at least 60 μg of EC and TC for each sample was firstly treated to remove carbonates (same protocol as discussed in concentration measurement). Filter subsamples were then subjected to the NIOSH-5040 protocol for oxidation to CO_2_, and the produced CO_2_ was cryotrapped during the EC combustion phase (after online removal of water and halogen-containing gases through magnesium perchlorate and silver wool traps, respectively^2, 3, 25, 27, 35^). A putative effect of OC charring on the estimated EC isotope composition with this approach has been considered and evaluated in earlier investigations^14, 16^. The concentration measurement for EC were here also found to be quite similar between the acid and non-acid treatments, suggesting that such acid fumigation effects are very limited (Table S6), which is consistent with the earlier sensitivity test results for charring^14, 16^. The analytical method for isolation of WSOC to determine its carbon isotope composition has also been described previously^2, 25, 35^. Briefly, a filter area required for the subsequent ^13^C and ^14^C measurements was extracted in 10 ml of Milli-Q water. The extracts were freeze-dried and then successively redissolved in 150 μl of 1 M hydrochloric acid, in order to decarbonate the WSOC samples. The WSOC samples were then transferred into precombusted silver capsules and dried in the oven at 60 °C. Finally, the dried WSOC samples and EC and TC samples in flame-sealed glass ampules were analyzed for their natural ^14^C abundance and ^13^C/^12^C ratio using accelerator mass spectrometry (AMS) at the US-NSF NOSAMS Facility (Woods Hole, MA, USA), as described previously^2, 3, 13, 25, 27, 35^. The isotopic compositions (^13^C and ^14^C) of OC (=TC − EC) and WIOC (=OC − WSOC) were derived from WSOC, TOC, and EC data using an isotope mass balance equation similar to previous studies^2, 25^. These data are presented in the Supporting Information (Table S2).

Fractional contribution of radiocarbon-extinct fossil fuel sources (*f*_fossil_) versus contemporary biomass/biogenic sources (*f*_bio_) can be determined using the isotopic mass balance equation:

1 $${{\rm{\Delta }}}^{14}{{\rm{C}}}_{{\rm{sample}}}={{\rm{\Delta }}}^{14}{{\rm{C}}}_{{\rm{fossil}}}{f}_{{\rm{fossil}}}+{{\rm{\Delta }}}^{14}{{\rm{C}}}_{{\rm{bio}}}(1-{f}_{{\rm{fossil}}})$$

where, Δ^14^C_sample_ represents the measured radiocarbon content of a sample and Δ^14^C_fossil_ is ‒ 1000‰, since fossil carbon is completely depleted in radiocarbon. End members for contemporary radiocarbon Δ^14^C_bio_ falls between +50 and +225‰. In East Asia, biomass value of Δ^14^C_bio_ = + 112 ± 60‰ is suggested and adopted^3, 14^, converting to a variability of <5% in the resulting calculated fraction of biomass using Markov-Chain Monte Carlo (MCMC) techniques.

### Source apportionment

The dual-carbon isotope signatures of EC were used in combination with a MCMC approach to further constrain the relative contributions from three source classes: biomass (*f*_bio_), coal (*f*_coal_) and liquid fossil fuel (*f*_liq fossil_)^3^.

2 $$(\begin{array}{c}{{\rm{\Delta }}}^{14}{{\rm{C}}}_{{\rm{sample}}}\\ {{\rm{\delta }}}^{13}{{\rm{C}}}_{{\rm{sample}}}\\ 1\end{array})=(\begin{array}{ccc}{{\rm{\Delta }}}^{14}{{\rm{C}}}_{{\rm{bio}}} & {{\rm{\Delta }}}^{14}{{\rm{C}}}_{\mathrm{liq}\mathrm{fossil}} & {{\rm{\Delta }}}^{14}{{\rm{C}}}_{{\rm{coal}}}\\ {{\rm{\delta }}}^{13}{{\rm{C}}}_{{\rm{bio}}} & {{\rm{\delta }}}^{13}{{\rm{C}}}_{{\rm{liq}}{\rm{fossil}}} & {{\rm{\delta }}}^{13}{{\rm{C}}}_{{\rm{coal}}}\\ 1 & 1 & 1\end{array})\cdot \,(\begin{array}{c}{f}_{{\rm{bio}}}\\ {f}_{{\rm{liq}}{\rm{fossil}}}\\ {f}_{{\rm{coal}}}\end{array})\,$$

where *f* denotes the fractional contribution from a given source, sample denotes the value of the analyzed field sample and the other isotope-values are source signatures (‘bio’, ‘liq fossil’, and ‘coal’ corresponding to biomass/biogenic, liquid fossil fuel, and coal respectively). The last row ensures the mass-balance principle.

### The calculation of ∆^13^C-signature

The Δ^13^C-signature of WSOC or WIOC is defined as:

3 $${{\rm{\Delta }}}^{13}{\rm{C}}={{\rm{\delta }}}^{13}{{\rm{C}}}_{{\rm{observed}}}-{{\rm{\delta }}}^{13}{{\rm{C}}}_{\mathrm{source}-\mathrm{predicted}}$$

where δ^13^C_observed_ is the observed signature for WSOC or WIOC, and δ^13^C_source-predicted_ is the predicted source mixture, defined as:

4 $${{\rm{\delta }}}^{13}{{\rm{C}}}_{\mathrm{source}-\mathrm{predicted}}={{\rm{\delta }}}^{13}{{\rm{C}}}_{{\rm{bio}}}\cdot {f}_{{\rm{bio}}}+{{\rm{\delta }}}^{13}{{\rm{C}}}_{{\rm{liq}}{\rm{fossil}}}\cdot {f}_{\mathrm{liq}\mathrm{fossil}}+{{\rm{\delta }}}^{13}{{\rm{C}}}_{{\rm{coal}}}\cdot {f}_{{\rm{coal}}}$$

where the parameters are defined analogously as in Eq (2). The *f*_bio_ is calculated from the Δ^14^C-signature, and thus *f*_fossil_ (=*f*_liq fossil_ + *f*_coal_). However, the division between the two fossil sources is not known for WSOC and WIOC. The central feature of computing the ∆^13^C-signature is to estimate if the δ^13^C_observed_ is significantly different from δ^13^C_source-predicted_, thus implying influence of atmospheric processing. To estimate the δ^13^C_source-predicted_, we use the leased biased representation of the two fossil sources, and represent the fractional contributions by a uniform probability density function (pdf; U[0,1]). The pdf for the δ^13^C_source-predicted_ is then calculated by integrating over the endmember and fractional fossil contributions using Monte Carlo numerical analysis. The mean δ^13^C_source-predicted_ and the numerical spread (e.g., the standard deviation) can be derived from this pdf, allowing to compute the mean and the source spread of the ∆^13^C. If the ∆^13^C is larger than the expected numerical spread, we infer that the δ^13^C_observed_ for WSOC or WIOC is significantly influenced by atmospheric processing.

### Data availability

The observatory data that support the finding of this study will be publicly available in the Bolin Center Database website (http://bolin.su.se/data/).

## Electronic supplementary material

Supplementary Information

## References

[1] Huang R-J (2014). High secondary organic aerosol contribution to particulate pollution during haze events in China. Nature.

[2] Kirillova EN, Andersson A, Han J, Lee M, Gustafsson Ö (2014). Sources and light absorption of water-soluble organic carbon aerosols in the outflow from northern China. Atmos. Chem. Phys..

[3] Andersson A (2015). Regionally-varying combustion sources of the January 2013 severe haze events over eastern China. Environ. Sci. Technol..

[4] Ji D (2014). The heaviest particulate air-pollution episodes occurred in northern China in January, 2013: Insights gained from observation. Atmos. Environ..

[5] WHO Air Quality Guidelines; http://whqlibdoc.who.int/hq/2006/WHO_SDE_PHE_OEH_06.02_eng.pdf (2005).

[6] Chinese State Council. *Air Pollution Prevention and Control Action Plan* (http://www.gov.cn/zwgk/2013-09/12/content_2486773.htm (in Chinese), 2013).

[7] Xinhua News Agency, PM_2.5_: *Decrease by approximately 40% in 2020 compared to 2013* (http://news.xinhuanet.com/politics/2015-12/30/c_1117630976.htm (in Chinese), 2015).

[8] Lu Z, Zhang Q, Streets DG (2011). Sulfur dioxide and primary carbonaceous aerosol emission trends in China and India, 1996–2010. Atmos. Chem. Phys..

[9] Ramanathan V, Carmichael G (2008). Global and regional climate changes due to black carbon. Nat. Geosci..

[10] Bond TC (2013). Bounding the role of black carbon in the climate system: a scientific assessment. J. Geophy. Res. Atmos..

[11] Bond TC (2004). A technology-based global inventory of black and organic carbon emissions from combustion. J. Geophy. Res. Atmos..

[12] Zhao Y, Nielsen CP, Lei Y, McElroy MB, Hao J (2011). Quantifying the uncertainties of a bottom-up emission inventory of anthropogenic atmospheric pollutants in China. Atmos. Chem. Phys..

[13] Gustafsson Ö (2009). Brown carbon over South Asia: biomass or fossil fuel combustion?. Science.

[14] Chen B (2013). Source forensics of black carbon aerosols from China. Environ. Sci. Technol..

[15] Budhavant K (2015). Radiocarbon-based source apportionment of elemental carbon aerosols at two South Asian receptor observatories over a full annual cycle. Environ. Res. Lett..

[16] Li C (2016). Sources of black carbon to the Himalayan–Tibetan Plateau glaciers. Nat. Commun..

[17] Stocker, T. F. *et al*. Climate Change 2013: The physical Science Basis. Contribution of Working Group I to the Fifth Assessment Report of the Intergovernmental Panel on Climate Change. (Cambridge University Press, 2013).

[18] Gustafsson Ö, Ramanathan V (2016). Convergence on climate warming by black carbon aerosols. Proc. Natl Acad. Sci. USA.

[19] Laskin A (2015). Chemistry of Atmospheric Brown Carbon. Chem. Rev..

[20] Andreae MO, Geleneser A (2006). Black carbon or brown carbon? The nature of the light-absorbing carbonaceous aerosols. Atmos. Chem. Phys..

[21] Alexander DTL (2008). Brown carbon spheres in East Asia outflow and their optical properties. Science.

[22] Feng Y, Ramanathan V, Kotamarthi VR (2013). Brown carbon: A significant atmospheric absorber of solar radiation?. Atmos. Chem. Phys..

[23] Chung CE (2012). Observational constrained estimates of carbonaceous aerosol radiative forcing. Proc. Natl. Acad. Sci. USA.

[24] Hecobian A (2010). Water-soluble organic carbon material and the light-absorption characteristic of aqueous extracts measured over the Southeastern United States. Atmos. Chem. Phys..

[25] Bosch C (2014). Source-diagnostic dual-isotope composition and optical properties of water-soluble organic carbon and elemental carbon in the South Asian outflow intercepted over the Indian Ocean. J. Geophy. Res. Atmos..

[26] Cheng Y (2011). Mass absorption efficiency of elemental carbon and water-soluble organic carbon in Beijing, China. Atmos. Chem. Phys..

[27] Winiger P, Andersson A, Eckhardt S, Stohl A, Gustafsson Ö (2016). The sources of atmospheric black carbon at a European gateway to the Arctic. Nat. Comm..

[28] Zhang YL (2015). Source apportionment of elemental carbon in Beijing, China: insights from radiocarbon and organic markers measurements. Environ. Sci. Technol..

[29] Liu D (2013). The use of levoglucosan and radiocarbon for source apportionment of PM_2.5_ carbonaceous aerosols at a background site in East China. Environ. Sci. Technol..

[30] Draxler, R. R. & Rolph, G. D. HYSPLIT (HYbrid Single-Particle Lagrangian Integrated Trajectory) Model access via NOAA ARL READY Website (http://www.arl.noaa.gov/ready/hysplit4.html). NOAA Air Resources Laboratory, Silver Spring, MD.

[31] Yan CQ, Zheng M (2015). Chemical characteristics and light-absorbing property of water-soluble organic carbon in Beijing: Biomass burning contributions. Atmos. Environ..

[32] Feng J (2006). A comparative study of the organic matter in PM_2.5_ from three Chinese megacities in three different climatic zones. Atmos. Environ..

[33] Huang H (2012). Characteristics of carbonaceous aerosol in PM_2.5_: Pearl Delta River Region, China. Atmos. Res..

[34] Zhang YL (2015). Fossil vs. non-fossil sources of fine carbonaceous aerosols in four Chinese cities during the extreme winter haze episode of 2013. Atmos. Chem. Phys..

[35] Kirillova EN (2014). Water-soluble organic carbon aerosols during a full New Delhi winter: Isotope-based source apportionment and optical properties. J. Geophy. Res. Atmos..

[36] Szidat S (2004). Source apportionment of aerosols by C-14 measurements in different carbonaceous particle fraction. Radiocarbon.

[37] Weber RJ (2007). A study of secondary organic aerosol formation in the anthropogenic-influences southeastern United States. J. Geophy. Res. Atmos..

[38] Wozniak AS (2012). Characteristics of water-soluble organic carbon associated with aerosol particles in the eastern United States. Atmos. Environ..

[39] Yan CQ, Zheng M (2017). Important fossil source contribution to brown carbon in Beijing during winter. Sci. Rep..

[40] Zheng M (2005). Seasonal trends in PM_2.5_ source contributions in Beijing, China. Atmos. Environ..

[41] Liu J (2016). Air pollutant emissions from Chinese households: A major and underappreciated ambient pollution source. Proc. Natl. Acad. Sci. USA.

[42] Ramana MV (2010). Warming influenced by the ratio of black carbon to sulphate and the black-carbon source. Nat. Geosci..

[43] Aggarwal SG, Kawamura K (2009). Carbonaceous and inorganic composition in long-range atmospheric transported aerosols over northern Japan: Implication for aging of water soluble organic fraction. Atmos. Environ..

[44] Irei S (2011). Stable carbon isotope ratio of secondary particulate organic matter formed by photooxidation of toluene in indoor smog chamber. Atmos. Environ..

[45] Aggarwal SG, Kawamura K (2008). Molecular distributions and stable carbon isotopic compositions of dicarboxylic acids and related compounds in aerosols from Sapporo, Japan: Implications for photochemical aging during long-range atmospheric transport. J. Geophy. Res. Atmos..

[46] Anderson RS (2004). Carbon kinetic isotope effects in the gas-phase reactions of aromatic hydrocarbons with the OH radical at 296 ± 4 K. Geophys. Res. Lett..

[47] Irei S (2015). Laboratory studies of carbon kinetic isotope effects on the production mechanism of particulate phenolic compounds formed by toluene photooxidation: A tool to constrain reaction pathways. J. Phys. Chem. A..

[48] Fisseha R (2009). Stable carbon isotope composition of secondary organic aerosol from beta-pinene oxidation. J. Geophy. Res. Atmos..

[49] Fang WZ (2011). Thermal desorption/Tunable vacuum-ultraviolet time-of-flight photoionization aerosol mass spectrometry for investigating secondary organic aerosols in chamber experiments. Anal. Chem..

[50] Fang WZ (2012). Measurements of secondary organic aerosol formed from OH-initiated photo-oxidation of isoprene using on-line photoionization aerosol mass spectrometry. Environ. Sci. Technol..

[51] Fang WZ (2017). Online analysis of secondary organic aerosols from OH-initiated photooxidation and ozonolysis of α-pinene, β-pinene, Δ^3^-carene and d-limonene by thermal desorption–photoionisation aerosol mass spectrometry. Environ. Chem..

[52] Kroll JH (2011). Carbon oxidation state as a metric for describing the chemistry of atmospheric organic aerosol. Nature Chemistry.

[53] Wang G (2010). Dicarboxylic acids, metals and isotopic compositions of C and N in atmospheric aerosols from inland China: Implications for dust and coal burning emission and secondary aerosol formation. Atmos. Chem. Phys..

[54] Elmquist M (2006). Distinct oxidative stabilities of char versus soot black carbon: Implications for quantification and environmental recalcitrance. Global Biogeochem. Cycles.

[55] Andreae MO, Rosenfeld D (2008). Aerosol-cloud-precipitation interactions. Part 1. The nature and sources of cloud-active aerosols. Earth Sci. Rev..

[56] Rudich Y, Donahue NM, Mentel TF (2007). Aging of organic aerosol: Bridging the gap between laboratory and field studies. Annu. Rev. Phys. Chem..

[57] Kalberer M (2004). Identification of polymers as major components of atmospheric organic aerosols. Science.

[58] Kim SW (2005). Aerosol optical, chemical, and physical properties at Gosan, Korea during Asian dust and pollution episodes in 2001. Atmos. Environ..

[59] Kim Y (2014). Aerosol properties and associated regional meteorology during winter pollution event at Gosan climate observatory, Korea. Atmos. Environ..

[60] Birch ME, Cary RA (1996). Elemental carbon-based method for monitoring occupational exposures to particulate diesel exhaust. Aerosol Sci. Technol..

