# Supplementary material for: Divergent Evolution of Carbonaceous Aerosols during Dispersal of East Asian Haze
Source: Sci Rep. 2017 Sep 5;7:10422. doi: 10.1038/s41598-017-10766-4 (PMC5585391; doi:10.1038/s41598-017-10766-4)
Supplement: Supplementary file 1 — Supplementary Information [file 41598_2017_10766_MOESM1_ESM.pdf]

Divergent Evolution of Carbonaceous Aerosols during Dispersal of East Asian Haze

Wenzheng Fang,[1] August Andersson,[1] Mei Zheng,[2] Meehye Lee,[3] Henry Holmstrand,[1] Sang-Woo Kim,[4] Ke Du,[5] & Örjan Gustafsson[1*]

[1]Department of Environmental Science and Analytical Chemistry (ACES) and Bolin Centre for Climate Research, Stockholm University, Stockholm 10691, Sweden

[2]College of Environmental Sciences and Engineering, Peking University, Beijing 100871, China

[3]Department of Earth and Environmental Sciences, Korea University, Seoul 02841, South Korea

[4]School of Earth and Environmental Sciences, Seoul National University, Seoul 08826, South Korea

[5]Department of Mechanical and Manufacturing Engineering, University of Calgary, Calgary, Canada T2N 1N4

* Correspondence and requests for materials should be addressed to Ö. G. (email: orjan.gustafsson@aces.su.se)

Supplementary Information Contents:

In total 35 pages including:

Figures: 23 figures (pages 3-26).

Tables: Five tables presenting data, source-endmember values, and field instrumentation (pages 27-33).

Text S1: Two regional sampling sites BTH and YRD (page 34).

Text S2. Back trajectories analysis (page 34).

Text S3. Acknowledgment (page 34).

References: References for the supplementary information (page 35).

Supplementary Figures

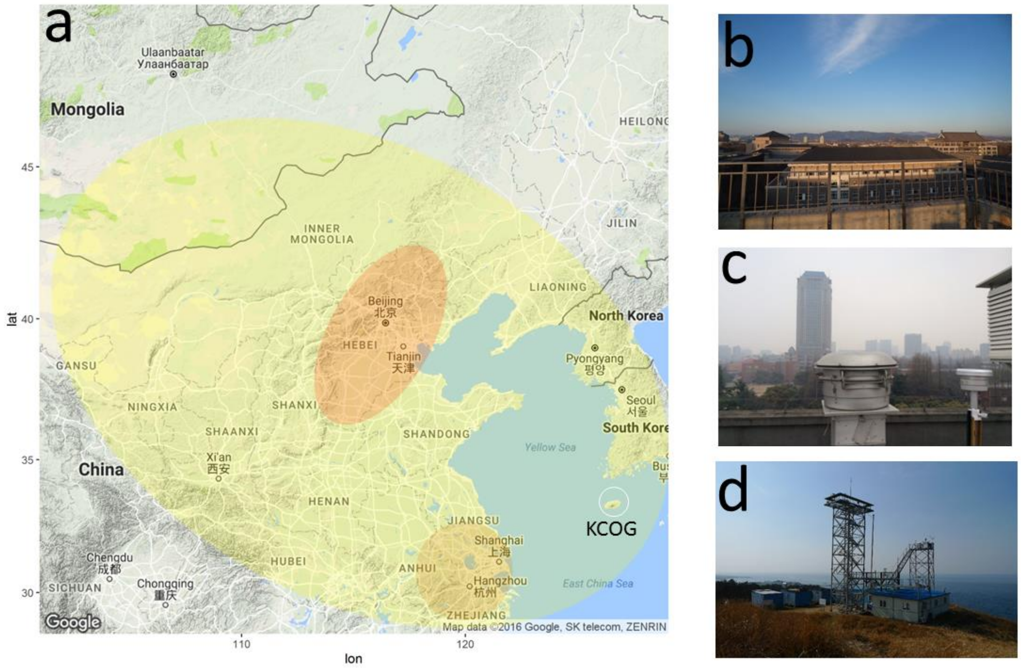

Figure S1. Sampling sites in detail. (a) Two urban sampling sites (megacity) Beijing and Shanghai, the two largest cities in North China Plain (NCP) and Yangtze River Delta (YRD), respectively. These are marked in red (Beijing-Tianjin-Hebei, BTH, part of the NCP) and orange (part of YRD) shaded area, respectively. The regional receptor sampling site Wuqing district (N39.38, E117.11) at Tianjin City is approximately 100 km southeast of Beijing. Wuqing is an ideal regional receptor site (downwind of main prevailing wind directions from the northern China) which is regarded as a representative atmosphere of Beijing-Tianjin-Hebei (BTH; is a part of NCP, red shaded area) region. Haining City (N30.51, E120.69) from Zhejiang province is a regional receptor site of YRD, located about 100 km southwest of Shanghai. The white circle denotes the Korea Climate Observatory at Gosan (KCOG, 72 m above sea level (a.s.l), N33.29, E126.16), an ideal location to intercept continental outflow

from East Asia[1], by the yellow-shaded area. Map of Fig. 1a was created by R (3.3) using Google maps.

(b) Photo of the Peking University sampling site (N39.59, E116.18), situated at Haidian district in the northwestern of Beijing (Photo by Mei Zheng, Peking University), as a representative urban atmosphere in Beijing, with no major emission sources in the immediate vicinity except two major roads, 150 m to the east and 200 m to the south. The sampling inlet was about 20 m above ground.

(c) Photo of the sampling site at the campus of Fudan University (N31.30, E121.50) in the northeast area of Shanghai (Photo by Mei Zheng, Peking University). This site is also located in an educational, commercial and residential district, with no obvious emission sources around except one major road about 50m away in the south. The sampling inlet was about 30 m above ground.

(d) Photo of the Southeast Yellow Sea recipient sampling site of KCOG on Jeju Island, South Korea (Photo by Örjan Gustafsson, Stockholm University). KCOG is located in a rural area at the western tip of Jeju Island, 100 km south of the Korean Peninsula, 500 km east of China, 200 km west of Kyushu Island. The sampling site is far away from the local residential area of this island and has been used in the many campaigns and programs such as the Gosan Pollution Experiment (GoPoEx)[2], the Aerosol Characterization Experiments (ACE) Asia[3], the Advanced Global Atmospheric Gases Experiment (AGAGE, http://agage.eas.gatech.edu/), the Global Atmosphere Watch (GAW) program (www.wmo.int/pages/prog/arep/gaw_home_en.html), and the International Atmospheric Brown Cloud (ABC) project (www.rrcap.unep.org/abc/). Meteorological data measurements at Gosan: Local meteorological measurements, including temperature, relative humidity, wind speed and direction at ground level, were continuously measured at Gosan weather station, maintained by the Korea Meteorological Administration (KMA).

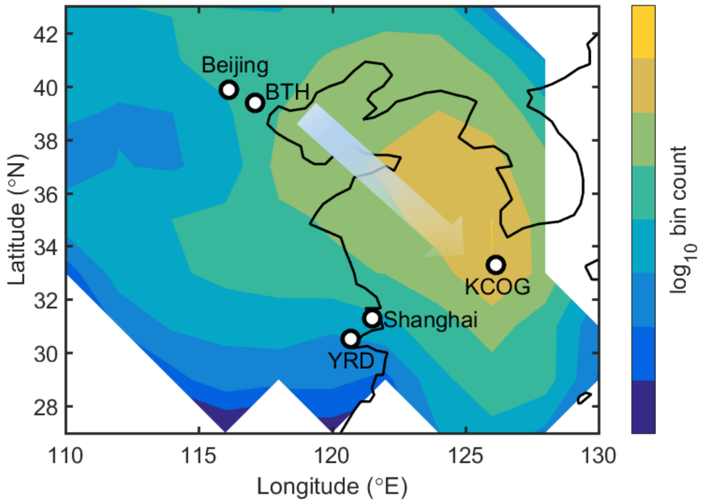

Figure S2. Probability density function of BTs for KCOG (Gosan on Jeju Island, South Korea) during January 2014. It indicates that prevailing air mass transport from Northeast Asia continent, as shown by the shaded arrow. Altitudes <1000 m are counted during air mass back-trajectories. The black and white circles denote locations of sampling sites: YRD (Haining, Zhejiang, China), BTH (Wuqing, Tianjin, China), Beijing (China), Shanghai (China), and KCOG (Gosan on Jeju Island, South Korea). The figure was created by MATLAB version R2015b (The MathWorks, Natick, MA, USA). Five-day air mass back-trajectories (BTs) for KCOG starting at 500 m height were calculated by NOAA HYSPLIT[4-6]. HYSPLIT: hybrid single particle Lagrangian Integrated trajectory model.

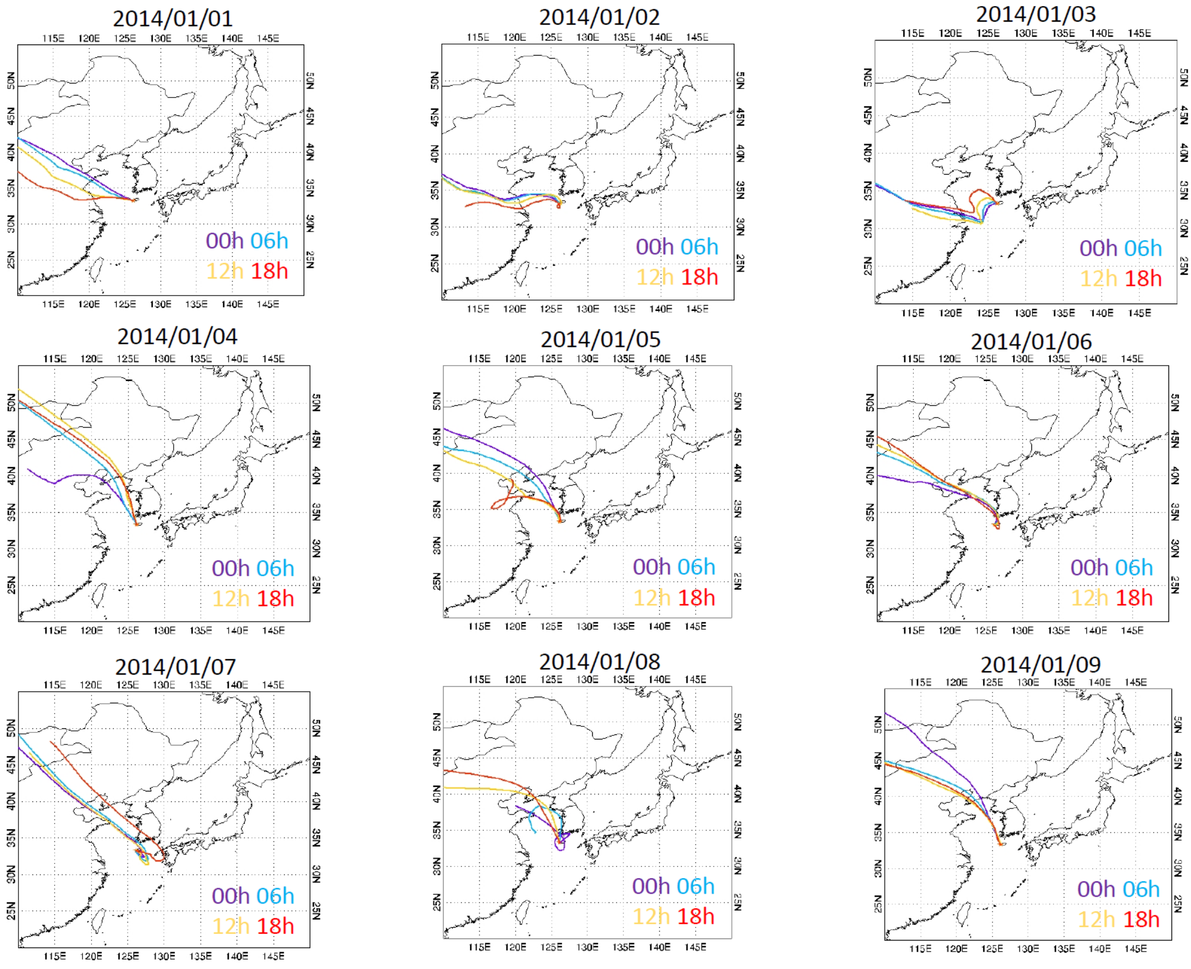

Figure S3. NOAA HYSPLIT[4-6] five-day air mass back-trajectories (BTs) for KCOG (Gosan on Jeju Island, South Korea) starting at 500 m height, every 6-hour during a day from 2014/01/01 to 2014/01/09. IDL (Version 7.1, 2009), a product of Exelis Visual Information Solutions, Inc., a subsidiary of Harris Corporation (Exelis VIS), was used to generate these maps. The Exelis VIS website can be found at http://www.exelisvis.com.

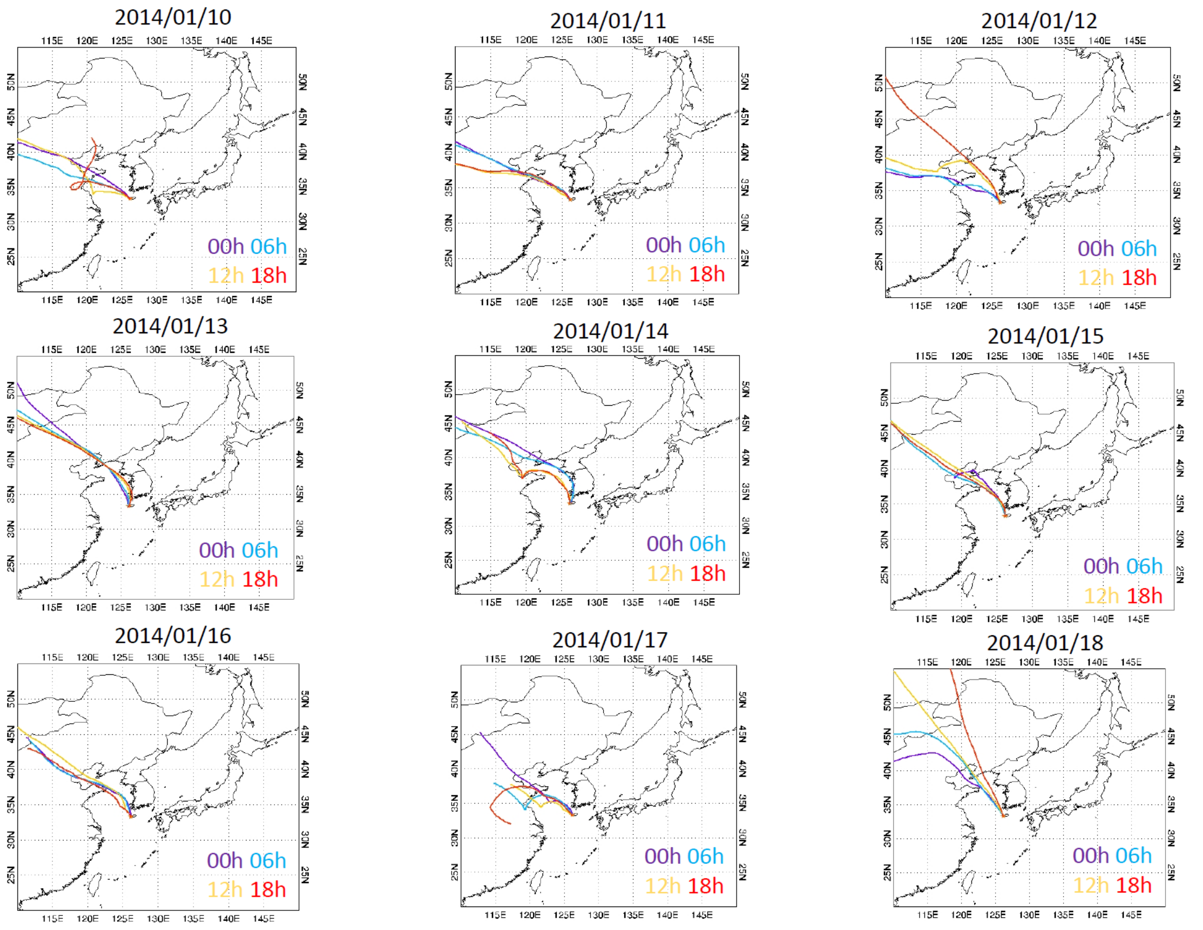

Figure S4. NOAA HYSPLIT[4-6] five-day air mass back-trajectories (BTs) for KCOG (Gosan on Jeju Island, South Korea) starting at 500 m height, every 6-hour during a day from 2014/01/10 to 2014/01/18. IDL (Version 7.1, 2009), a product of Exelis Visual Information Solutions, Inc., a subsidiary of Harris Corporation (Exelis VIS), was used to generate these maps. The Exelis VIS website can be found at http://www.exelisvis.com.

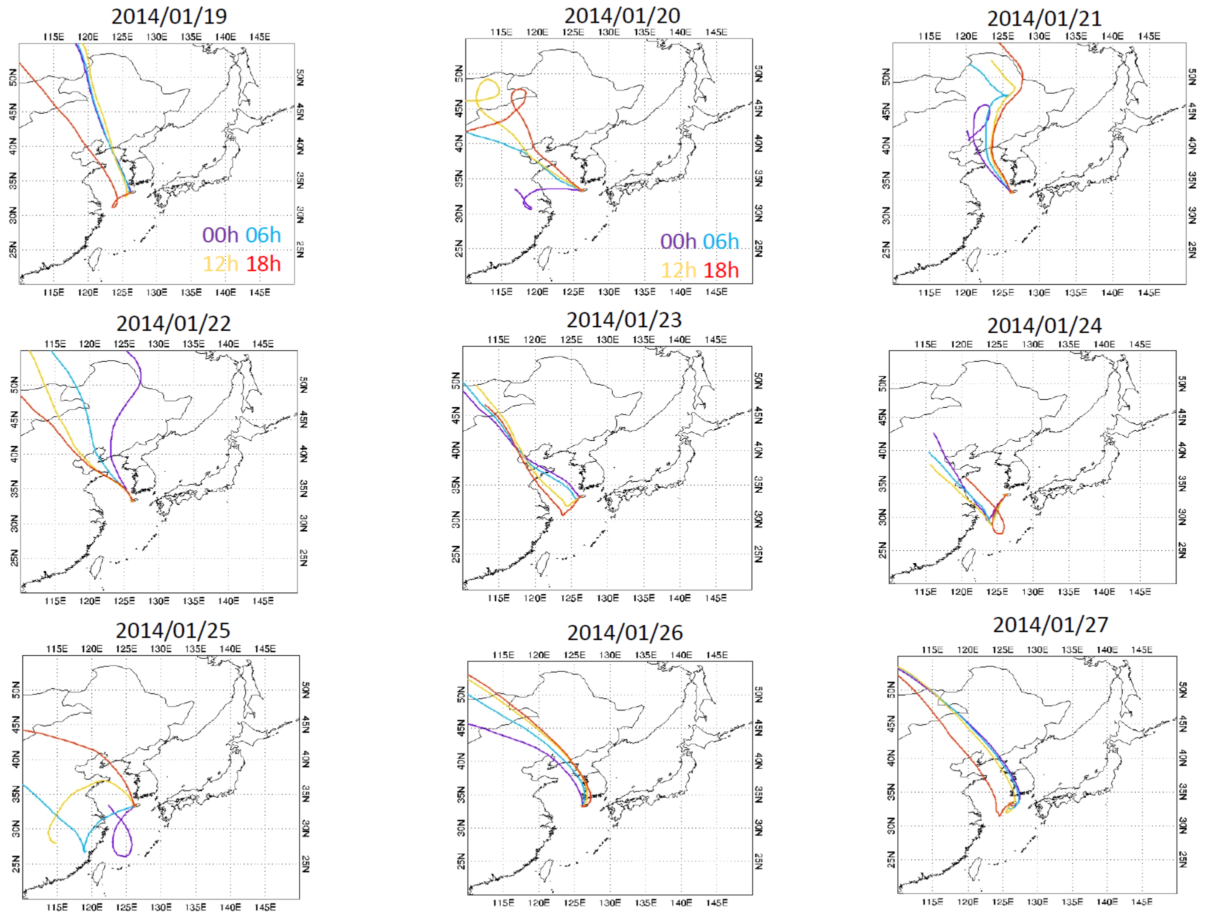

Figure S5. NOAA HYSPLIT[4-6] five-day air mass back-trajectories (BTs) for KCOG (Gosan on Jeju Island, South Korea) starting at 500 m height, every 6-hour during a day from 2014/01/19 to 2014/01/27. IDL (Version 7.1, 2009), a product of Exelis Visual Information Solutions, Inc., a subsidiary of Harris Corporation (Exelis VIS), was used to generate these maps. The Exelis VIS website can be found at http://www.exelisvis.com.

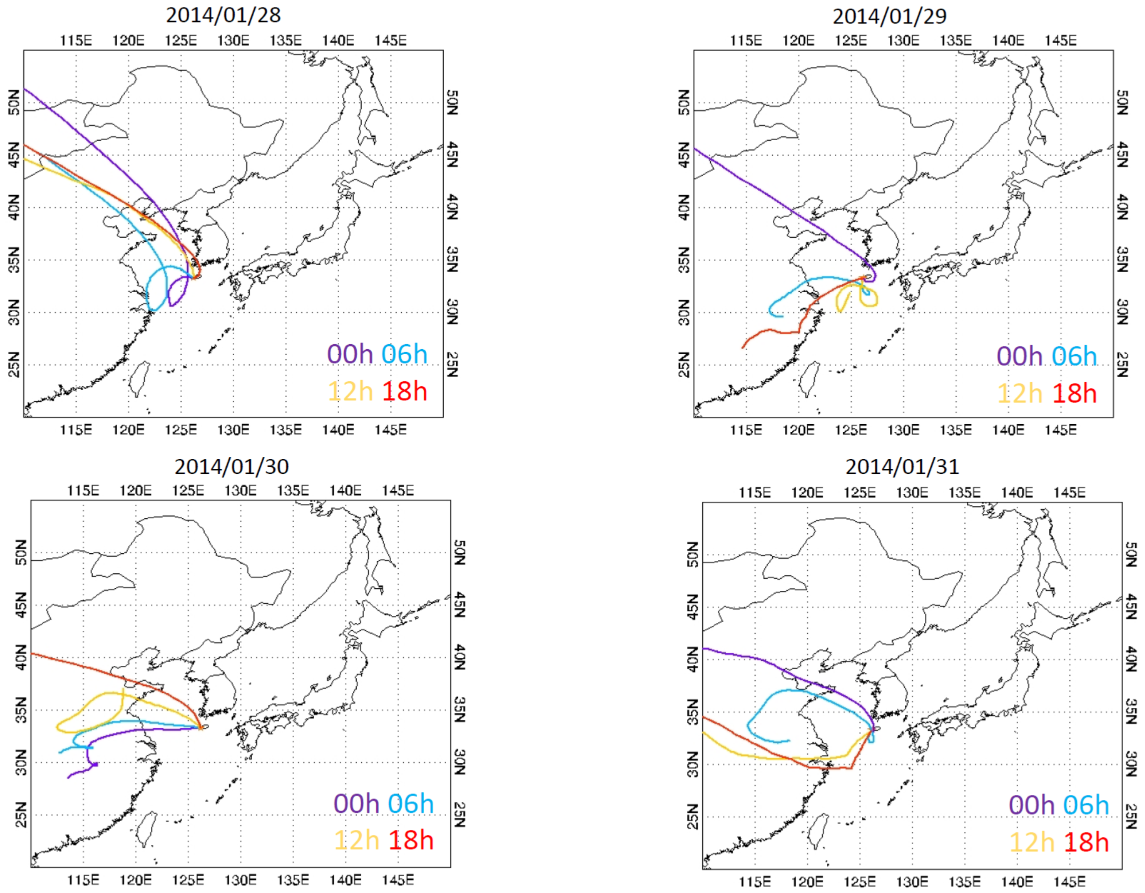

Figure S6. NOAA HYSPLIT[4-6] five-day air mass back-trajectories (BTs) for KCOG (Gosan on Jeju Island, South Korea) starting at 500 m height, every 6-hour during a day from 2014/01/28 to 2014/01/31. IDL (Version 7.1, 2009), a product of Exelis Visual Information Solutions, Inc., a subsidiary of Harris Corporation (Exelis VIS), was used to generate these maps. The Exelis VIS website can be found at http://www.exelisvis.com.

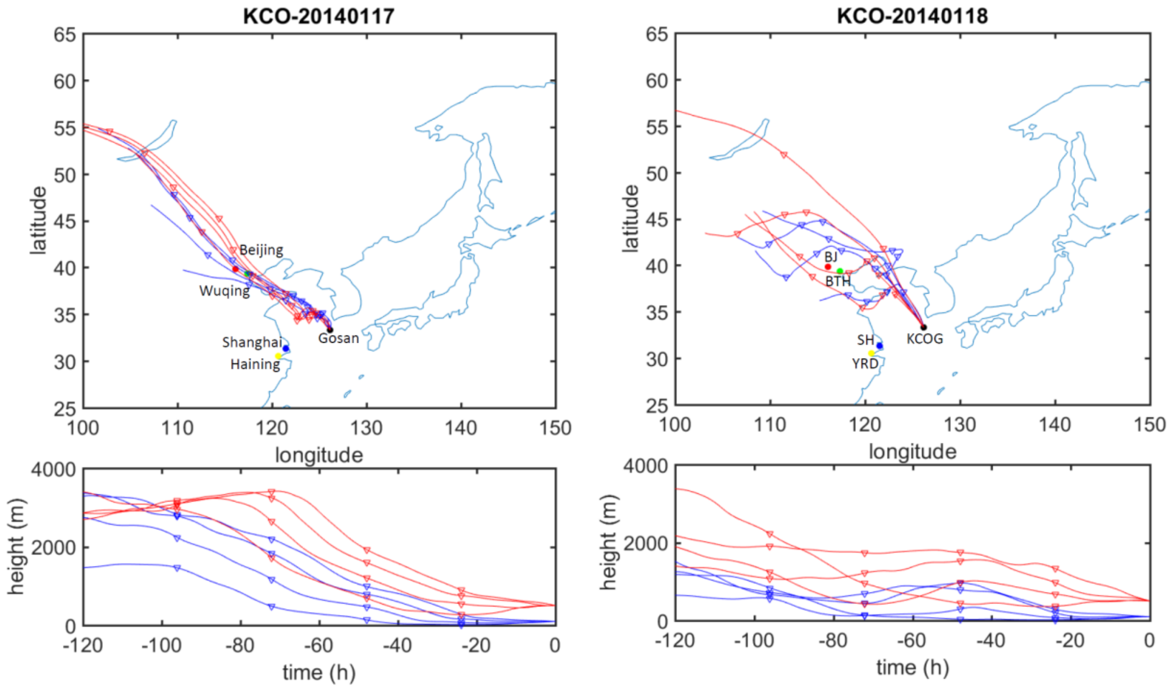

Figure S7. NOAA HYSPLIT[4-6] five-day air mass back-trajectories (BTs) for KCOG (Gosan on Jeju Island, South Korea) starting at heights of 100 and 500 m, every 6-hour at each starting height during a day for 2014/01/17 and 2014/01/18. BJ, BTH, SH, YRD, and KCOG denote Beijing, Wuqing (Tianjin, China), Shanghai, Haining (China) and Korea Climate Observatory at Gosan, respectively. The figure was created by MATLAB version R2015b (The MathWorks, Natick, MA, USA).

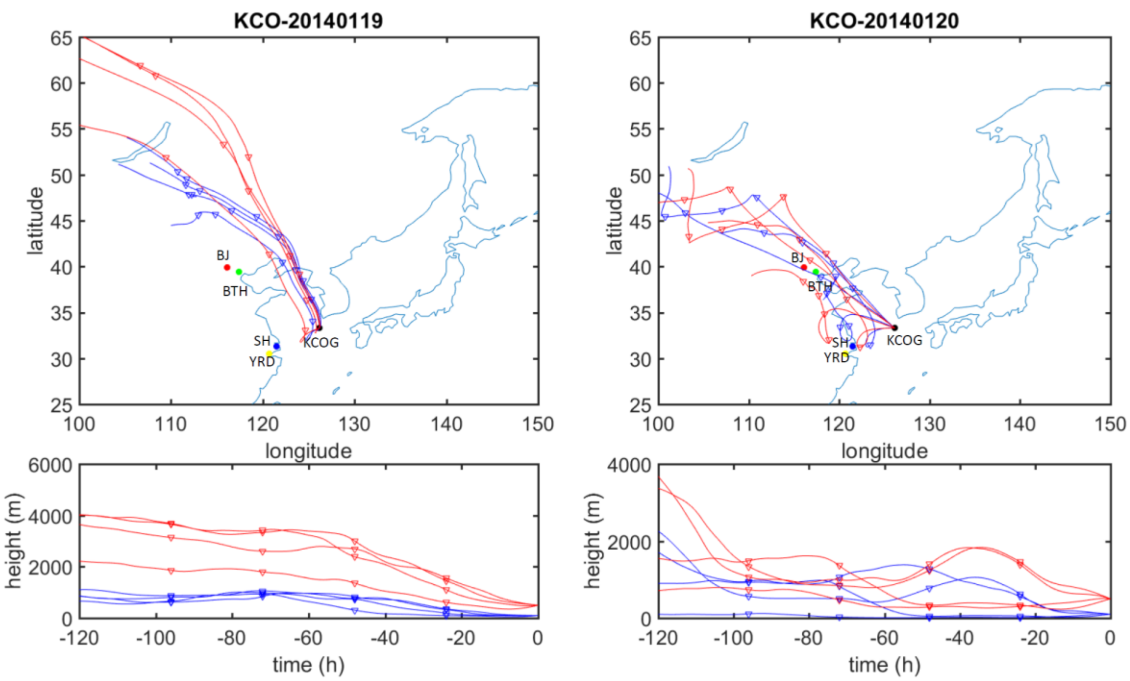

Figure S8. NOAA HYSPLIT[4-6] five-day air mass back-trajectories (BTs) for KCOG (Gosan on Jeju Island, South Korea) starting at heights of 100 and 500 m, every 6-hour at each starting height during a day for 2014/01/19 and 2014/01/20. BJ, BTH, SH, YRD, and KCOG denote Beijing, Wuqing (Tianjin, China), Shanghai, Haining (China) and Korea Climate Observatory at Gosan, respectively. The figure was created by MATLAB version R2015b (The MathWorks, Natick, MA, USA).

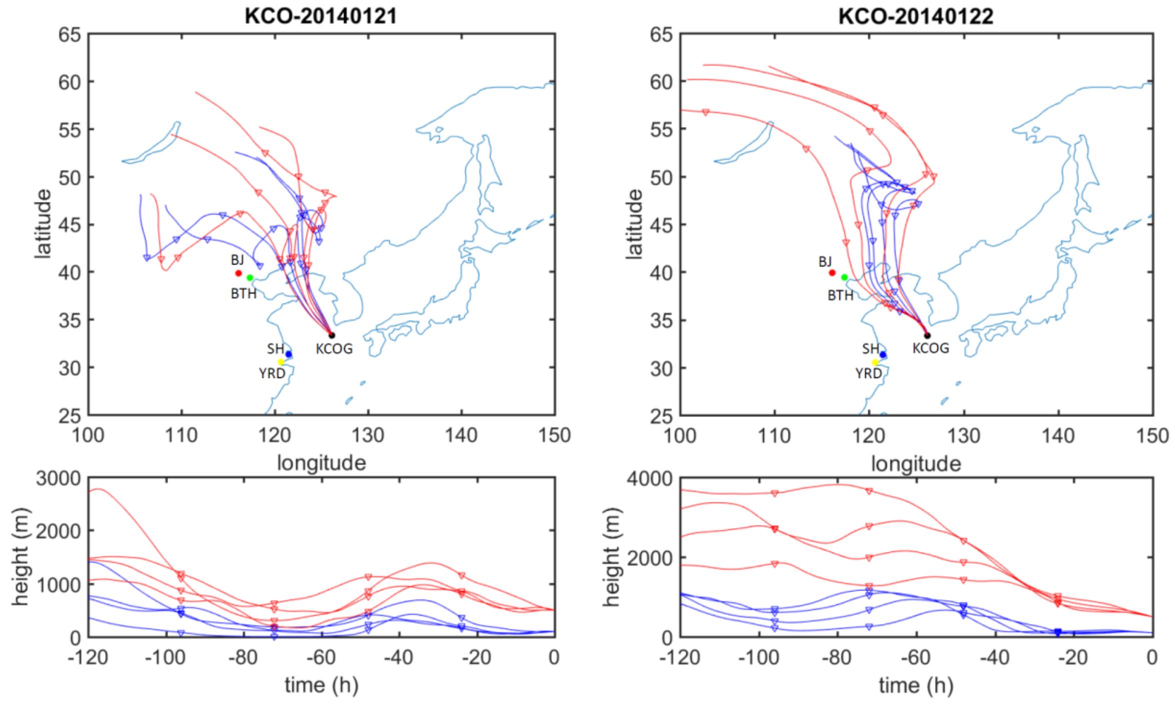

Figure S9. NOAA HYSPLIT[4-6] five-day air mass back-trajectories (BTs) for KCOG (Gosan on Jeju Island, South Korea) starting at heights of 100 and 500 m, every 6-hour at each starting height during a day for 2014/01/21 and 2014/01/22. BJ, BTH, SH, YRD, and KCOG denote Beijing, Wuqing (Tianjin, China), Shanghai, Haining (China) and Korea Climate Observatory at Gosan, respectively. The figure was created by MATLAB version R2015b (The MathWorks, Natick, MA, USA).

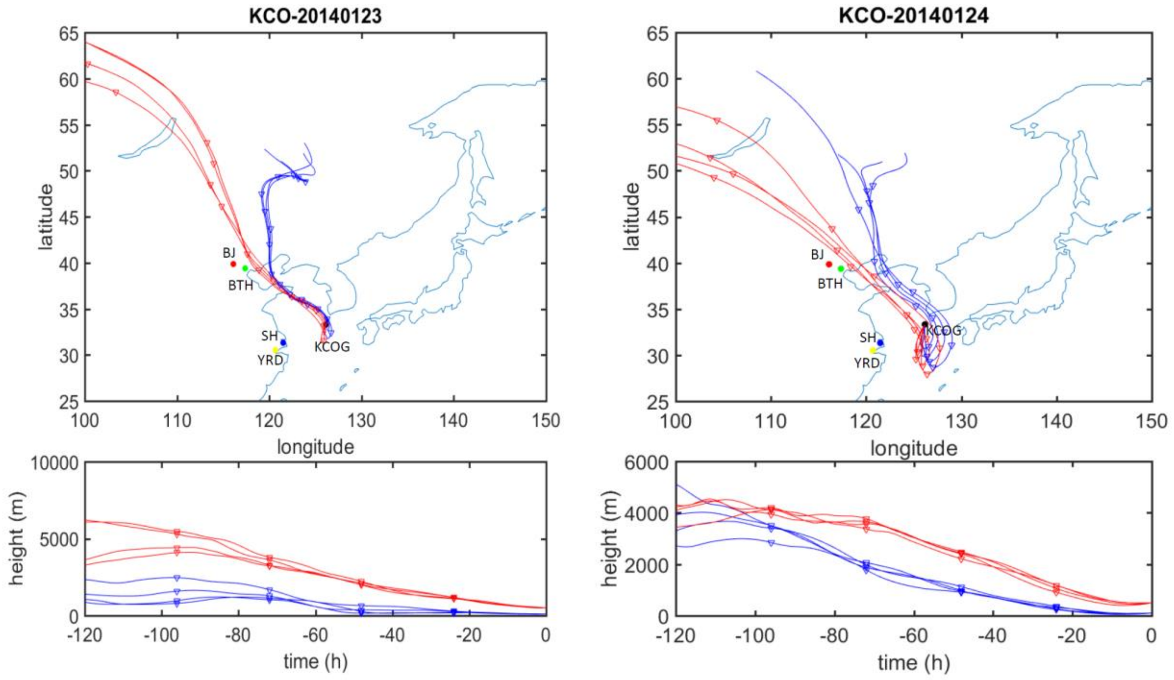

Figure S10. NOAA HYSPLIT[4-6] five-day air mass back-trajectories (BTs) for KCOG (Gosan on Jeju Island, South Korea) starting at heights of 100 and 500 m, every 6-hour at starting each height during a day for 2014/01/23 and 2014/01/24. BJ, BTH, SH, YRD, and KCOG denote Beijing, Wuqing (Tianjin, China), Shanghai, Haining (China) and Korea Climate Observatory at Gosan, respectively. The figure was created by MATLAB version R2015b (The MathWorks, Natick, MA, USA).

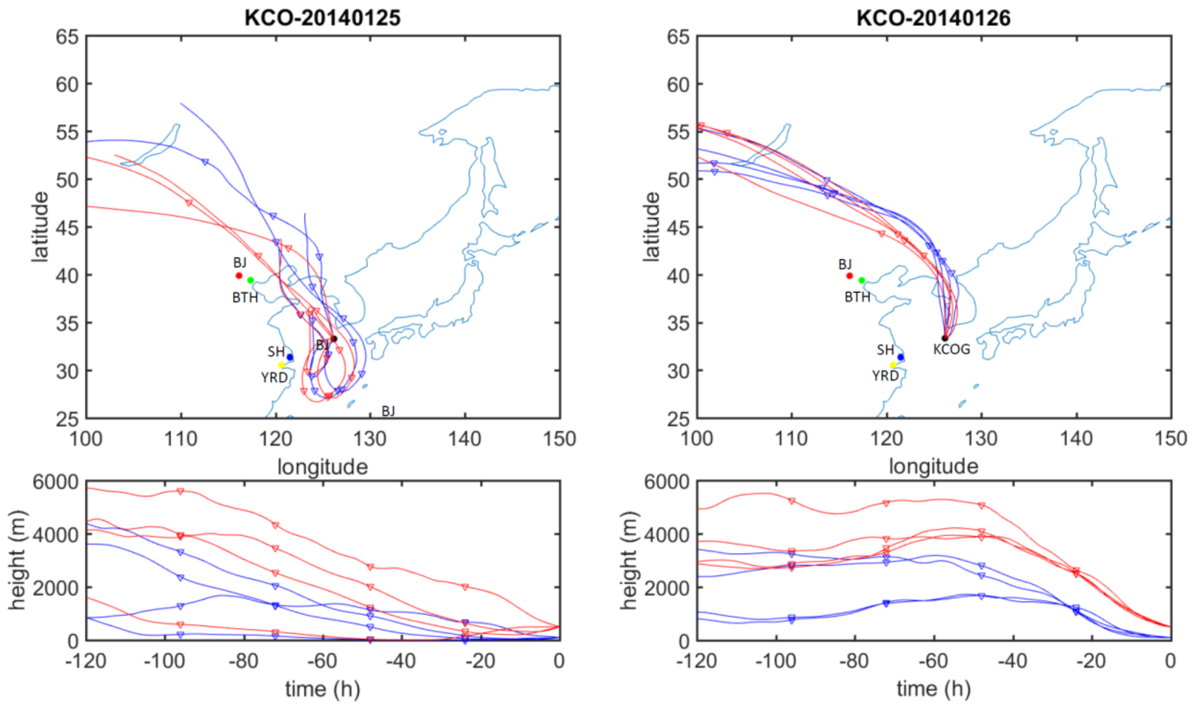

Figure S11. NOAA HYSPLIT[4-6] five-day air mass back-trajectories (BTs) for KCOG (Gosan on Jeju Island, South Korea) starting at heights of 100 and 500 m, every 6-hour at each starting height during a day for 2014/01/25 and 2014/01/26. BJ, BTH, SH, YRD, and KCOG denote Beijing, Wuqing (Tianjin, China), Shanghai, Haining (China) and Korea Climate Observatory at Gosan, respectively. The figure was created by MATLAB version R2015b (The MathWorks, Natick, MA, USA).

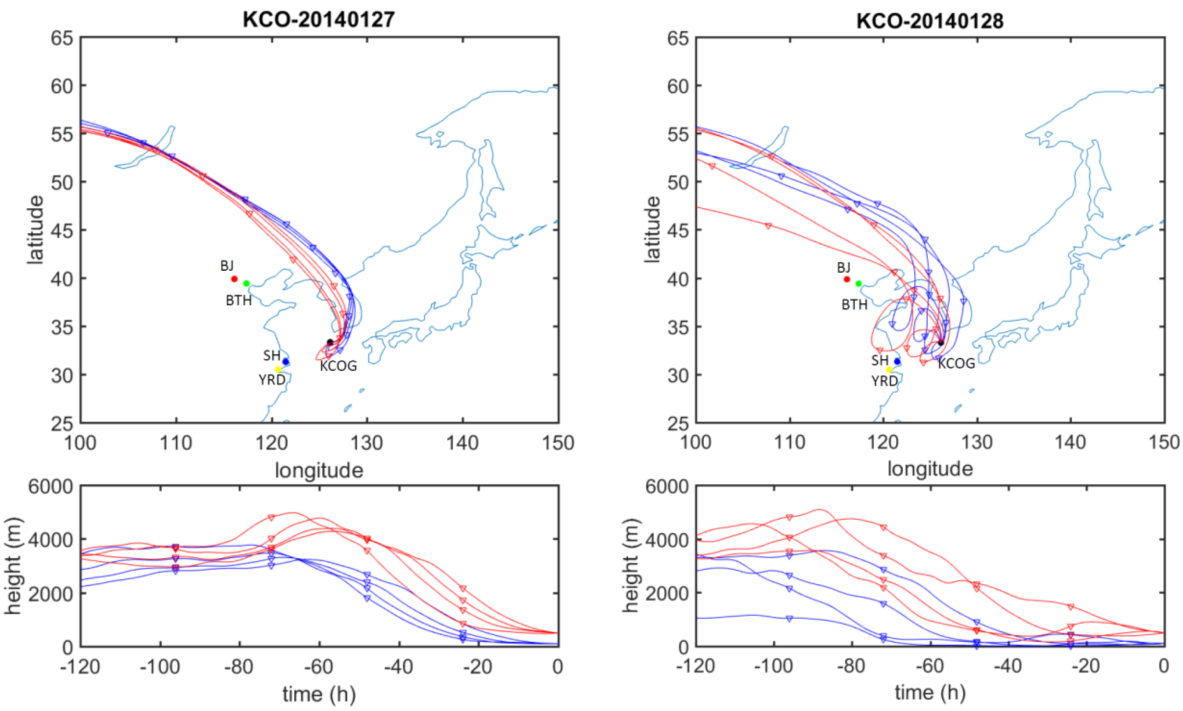

Figure S12. NOAA HYSPLIT[4-6] five-day air mass back-trajectories (BTs) for KCOG (Gosan on Jeju Island, South Korea) starting at heights of 100 and 500 m, every 6-hour at each starting height during a day for 2014/01/27 and 2014/01/28. BJ, BTH, SH, YRD, and KCOG denote Beijing, Wuqing (Tianjin, China), Shanghai, Haining (China) and Korea Climate Observatory at Gosan, respectively. The figure was created by MATLAB version R2015b (The MathWorks, Natick, MA, USA).

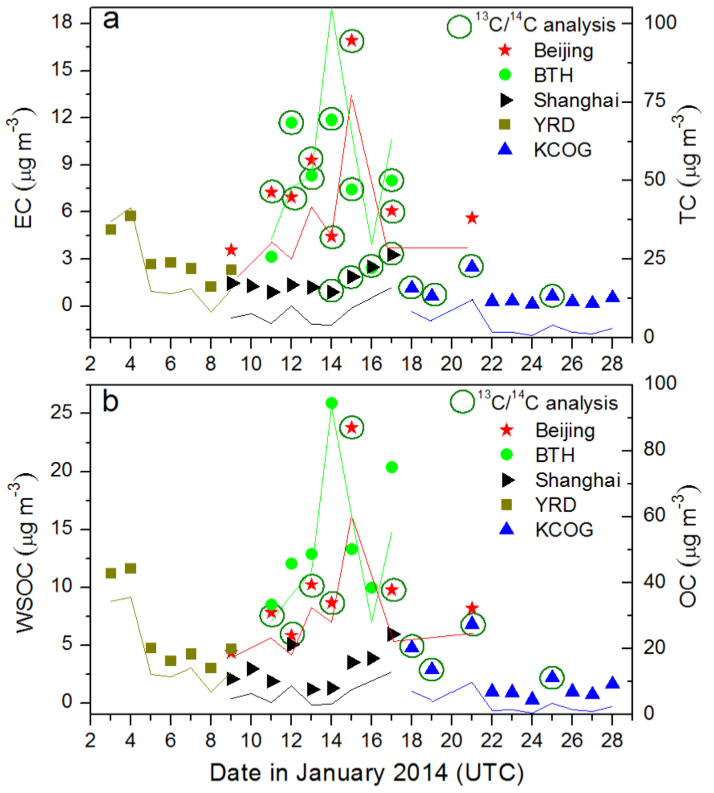

Figure S13. Temporal variations of EC, TC, WSOC, and OC concentrations in $PM_{2.5}$ samples and selected samples for dual-carbon isotope analysis over East Asia in January 2014. Olive circles denote EC (including Beijing, BTH, Shanghai, and KCOG), WSOC (including Beijing and KCOG) and TC (including Beijing and KCOG) isolates collected for stable carbon ($\delta^{13}C$) and radiocarbon ($\Delta^{14}C$) analysis. **(a)** Concentrations of EC (symbols, left) and TC (lines, right) at Beijing (red), Shanghai (black), and regional sites Beijing-Tianjin-Hebei (BTH, green), Yangtze River Delta (YRD, dark yellow), and Korea Climate Observatory at Gosan (KCOG, blue) during the sampling campaigns. **(b)** Concentrations of WSOC (symbols, left) and OC (lines, right) at Beijing (red), Shanghai (black), BTH (green), YRD (dark yellow), and KCOG (blue). Data points of 18th, 19th, 21th, and 25th January 2014 correspond to the sample ID of KCOG-0117, KCOG-0119, KCOG-0120, KCOG-0125, respectively. The sampling durations for these samples are given in Table S1.

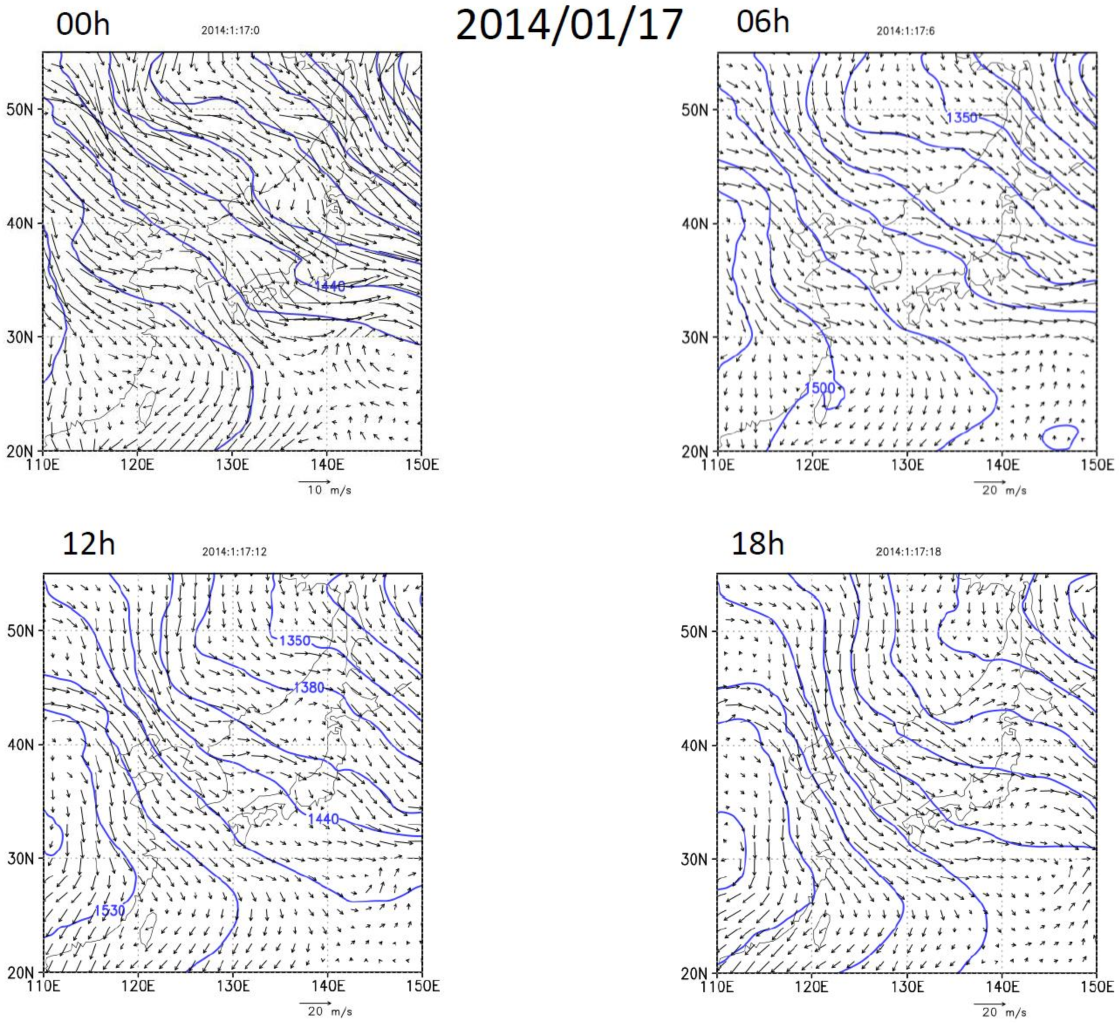

Figure S14. Synoptic weather pattern (geopotential height and wind vectors at 850-hPa pressure level) in East Asia on 2014/01/17 (every 6 hours). IDL (Version 7.1, 2009), a product of Exelis Visual Information Solutions, Inc., a subsidiary of Harris Corporation (Exelis VIS), was used to generate these maps. The Exelis VIS website can be found at http://www.exelisvis.com. The weather patterns were made with Grid Analysis and Display System (GrADS) Version 2.1.a3 © 1988-2015 and ERA Interim, daily data at 850 hPa were used from ECMWF.

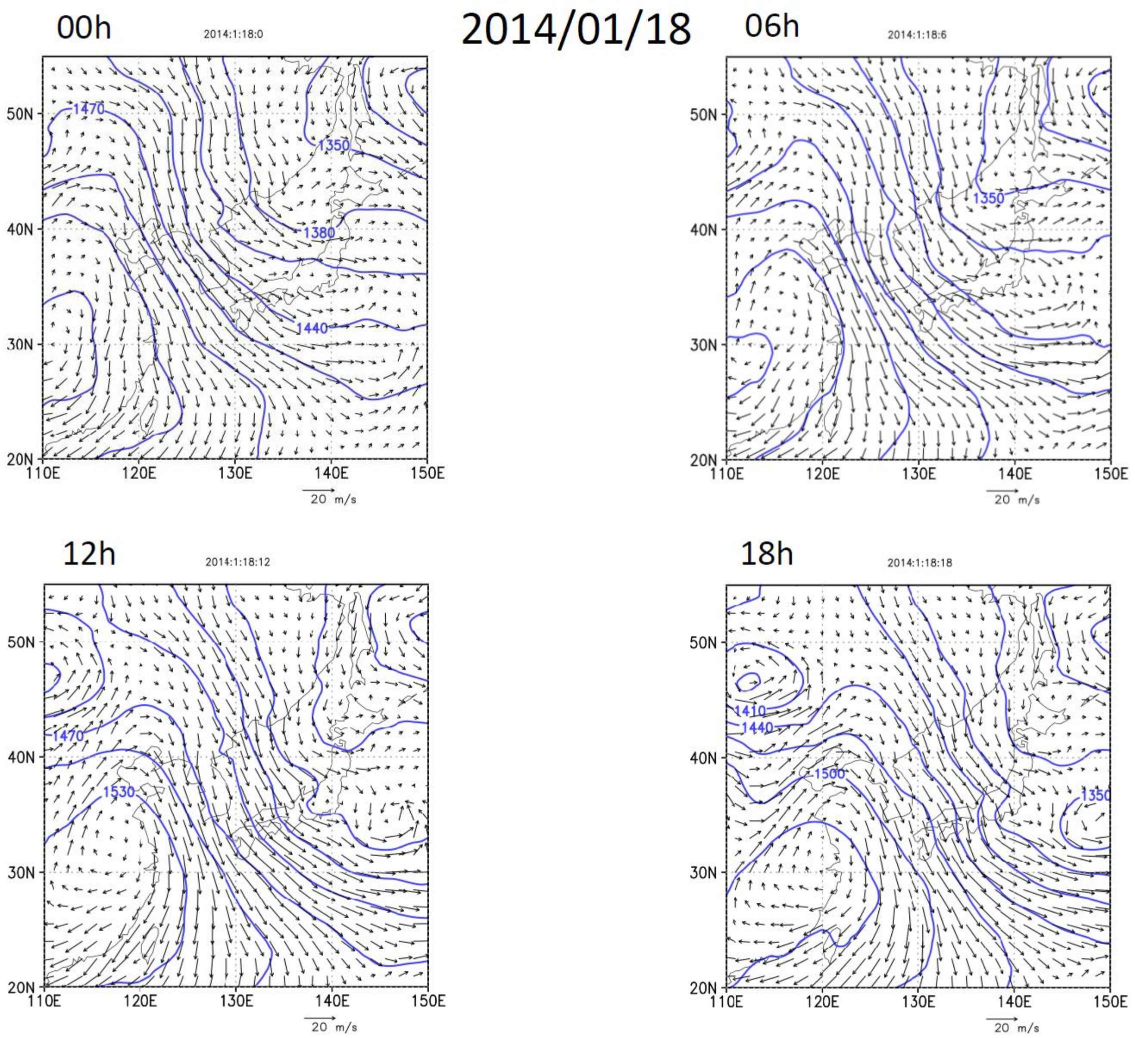

Figure S15. Synoptic weather pattern (geopotential height and wind vectors at 850-hPa pressure level) in East Asia on 2014/01/18 (every 6 hours). IDL (Version 7.1, 2009), a product of Exelis Visual Information Solutions, Inc., a subsidiary of Harris Corporation (Exelis VIS), was used to generate these maps. The Exelis VIS website can be found at http://www.exelisvis.com. The weather patterns were made with Grid Analysis and Display System (GrADS) Version 2.1.a3 © 1988-2015 and ERA Interim, daily data at 850 hPa were used from ECMWF.

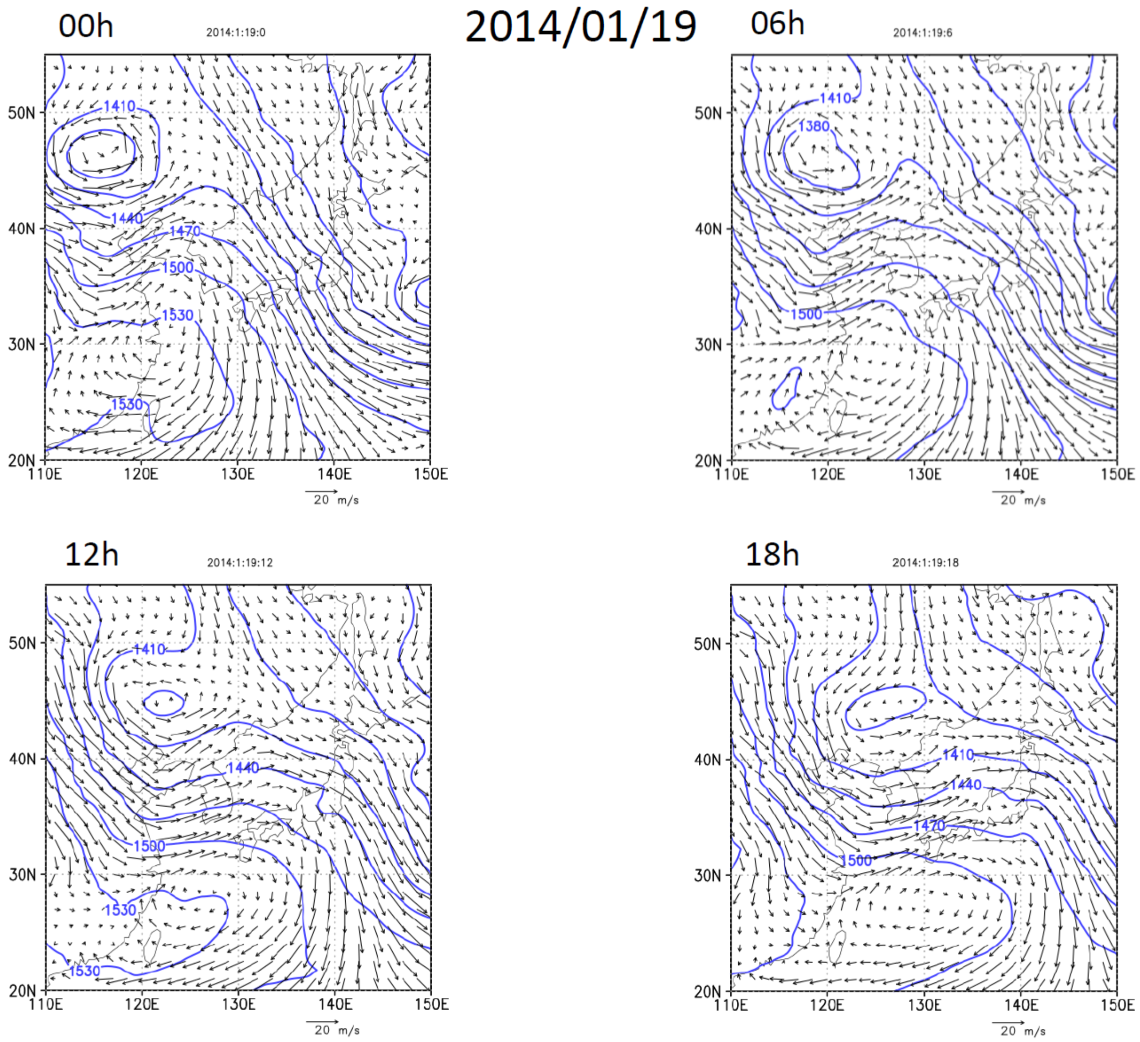

Figure S16. Synoptic weather pattern (geopotential height and wind vectors at 850-hPa pressure level) in East Asia on 2014/01/19 (every 6 hours). IDL (Version 7.1, 2009), a product of Exelis Visual Information Solutions, Inc., a subsidiary of Harris Corporation (Exelis VIS), was used to generate these maps. The Exelis VIS website can be found at http://www.exelisvis.com. The weather patterns were made with Grid Analysis and Display System (GrADS) Version 2.1.a3 © 1988-2015 and ERA Interim, daily data at 850 hPa were used from ECMWF.

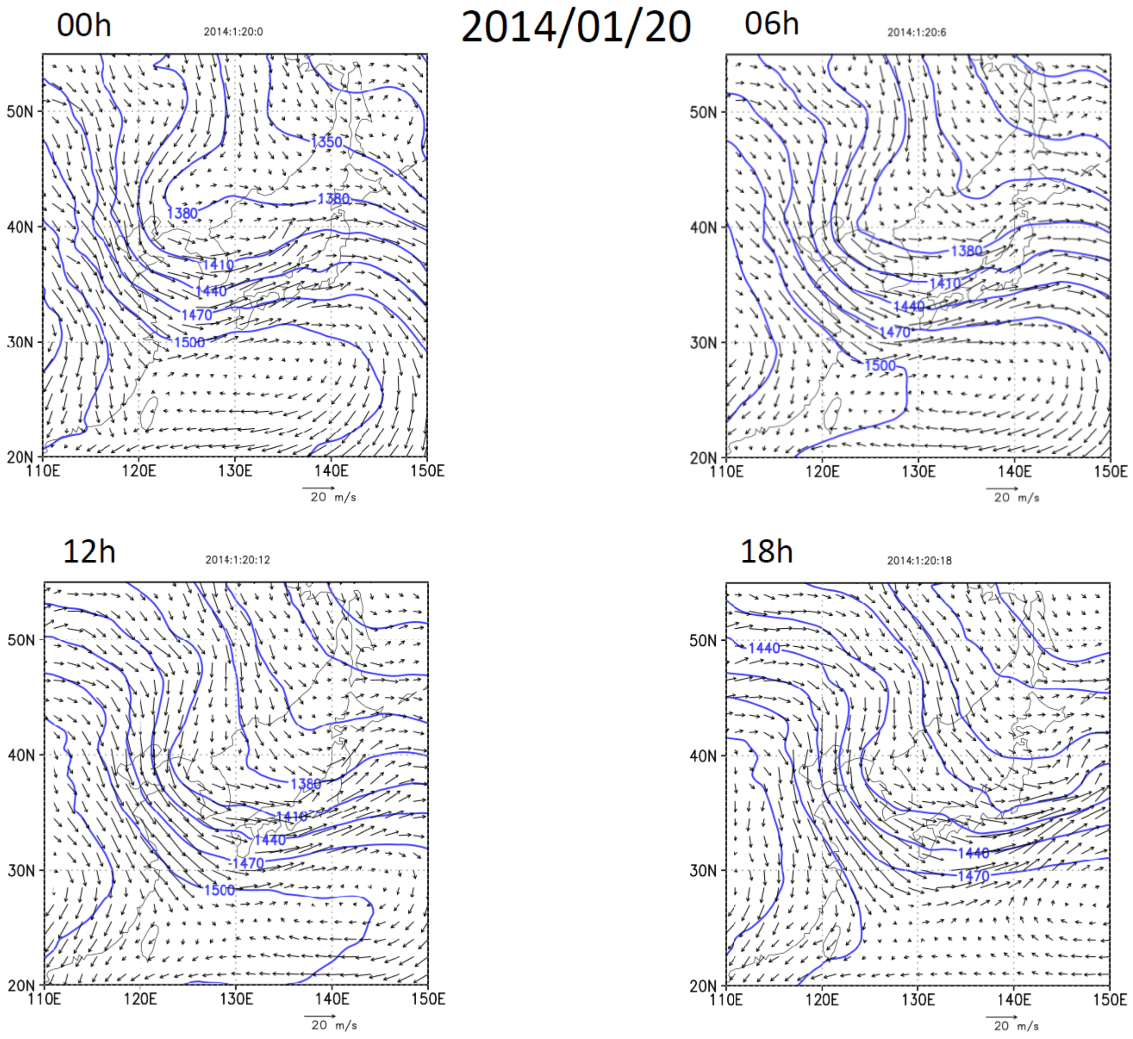

Figure S17. Synoptic weather pattern (geopotential height and wind vectors at 850-hPa pressure level) in East Asia on 2014/01/20 (every 6 hours). IDL (Version 7.1, 2009), a product of Exelis Visual Information Solutions, Inc., a subsidiary of Harris Corporation (Exelis VIS), was used to generate these maps. The Exelis VIS website can be found at http://www.exelisvis.com. The weather patterns were made with Grid Analysis and Display System (GrADS) Version 2.1.a3 © 1988-2015 and ERA Interim, daily data at 850 hPa were used from ECMWF.

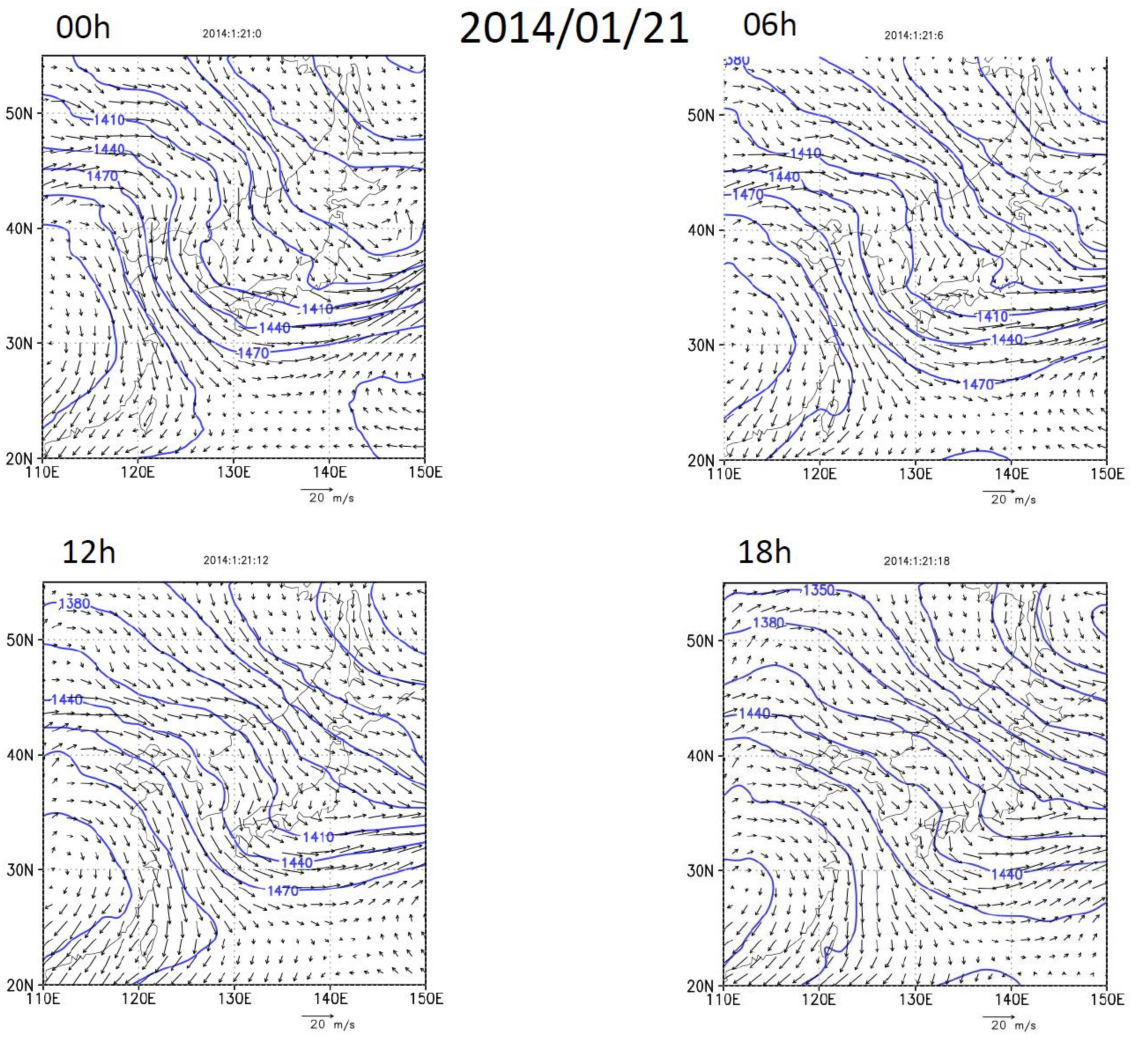

Figure S18. Synoptic weather pattern (geopotential height and wind vectors at 850-hPa pressure level) in East Asia on 2014/01/21 (every 6 hours). IDL (Version 7.1, 2009), a product of Exelis Visual Information Solutions, Inc., a subsidiary of Harris Corporation (Exelis VIS), was used to generate these maps. The Exelis VIS website can be found at http://www.exelisvis.com. The weather patterns were made with Grid Analysis and Display System (GrADS) Version 2.1.a3 © 1988-2015 and ERA Interim, daily data at 850 hPa were used from ECMWF.

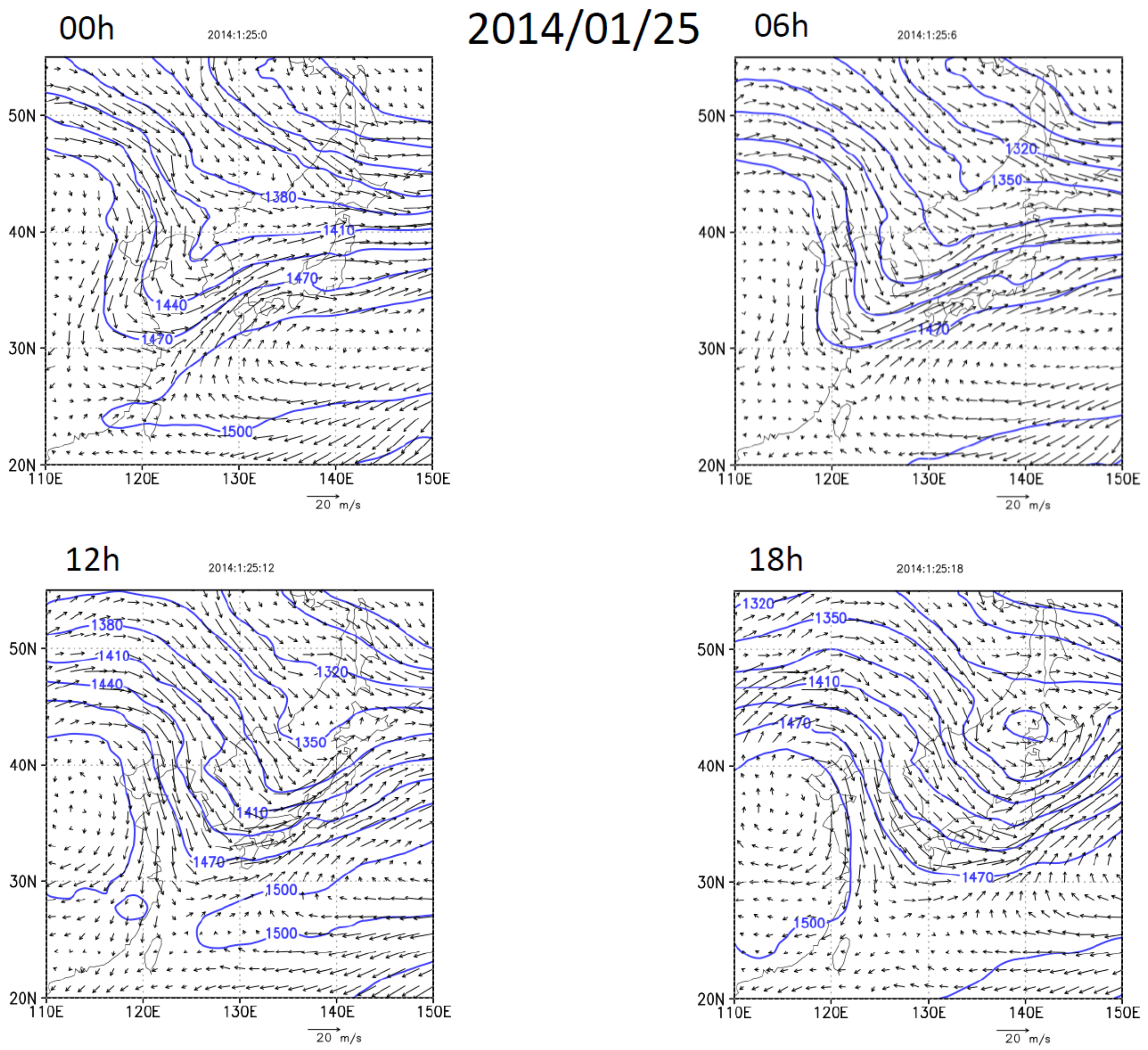

Figure S19. Synoptic weather pattern (geopotential height and wind vectors at 850-hPa pressure level) in East Asia on 2014/01/25 (every 6 hours). IDL (Version 7.1, 2009), a product of Exelis Visual Information Solutions, Inc., a subsidiary of Harris Corporation (Exelis VIS), was used to generate these maps. The Exelis VIS website can be found at http://www.exelisvis.com. The weather patterns were made with Grid Analysis and Display System (GrADS) Version 2.1.a3 © 1988-2015 and ERA Interim, daily data at 850 hPa were used from ECMWF.

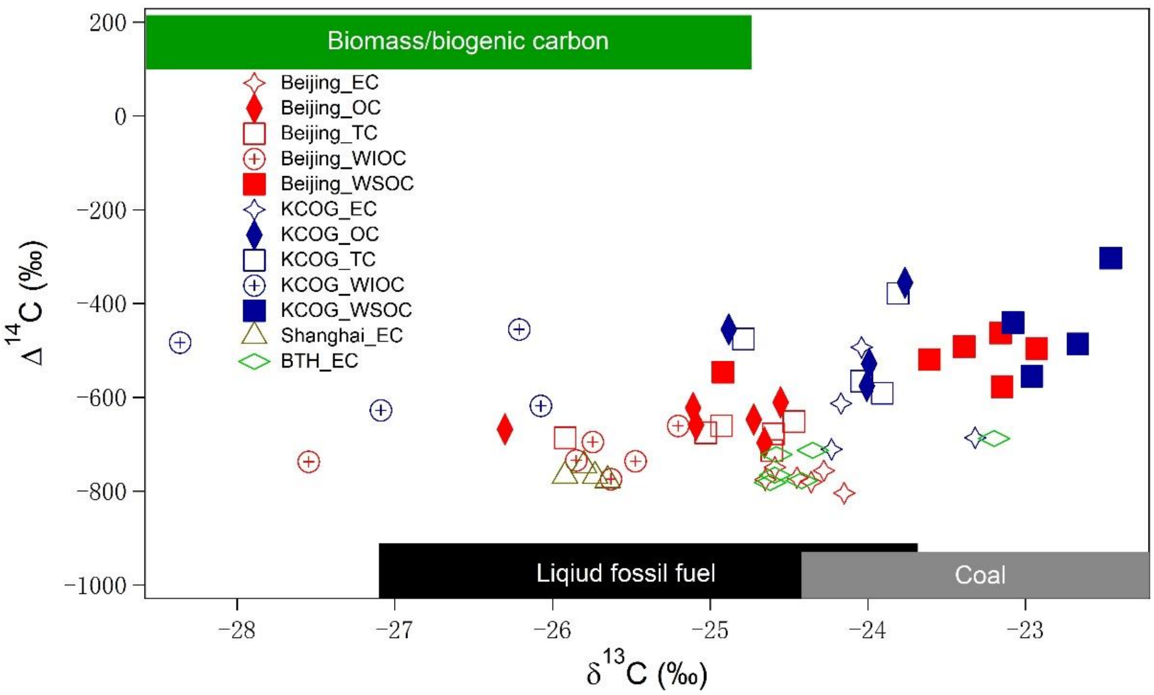

Figure S20. Two-dimensional dual carbon isotope $\delta^{13}C$ versus $\Delta^{14}C$ presentation of carbon aerosol fraction: (a) elemental carbon (EC, including Beijing-EC, Shanghai-EC, KCOG-EC, BTH-EC), **(b)** organic carbon (OC, including Beijing-OC & KCOG-OC), **(c)** total carbon (TC, including Beijing-TC & KCOG-TC), **(d)** water-soluble organic carbon (WSOC, including Beijing-WSOC & KCOG-WSOC), and **(e)** water-insoluble organic carbon (WSOC, including Beijing-WIOC & KCOG-WIOC) in $PM_{2.5}$ in January 2014 E. Asia campaign. Colored symbols indicate dual carbon signals of different carbon aerosol fractions in different locations. The $\delta^{13}C$ source-signature ranges (mean ± standard deviation) for the C3 plant, liquid fossil fuel, and coal are outlined by light shaded rectangles within the $\Delta^{14}C$-based endmember ranges for biomass combustion or biogenic source (green, top), liquid fossil fuel (black, bottom), and coal (grey, bottom).

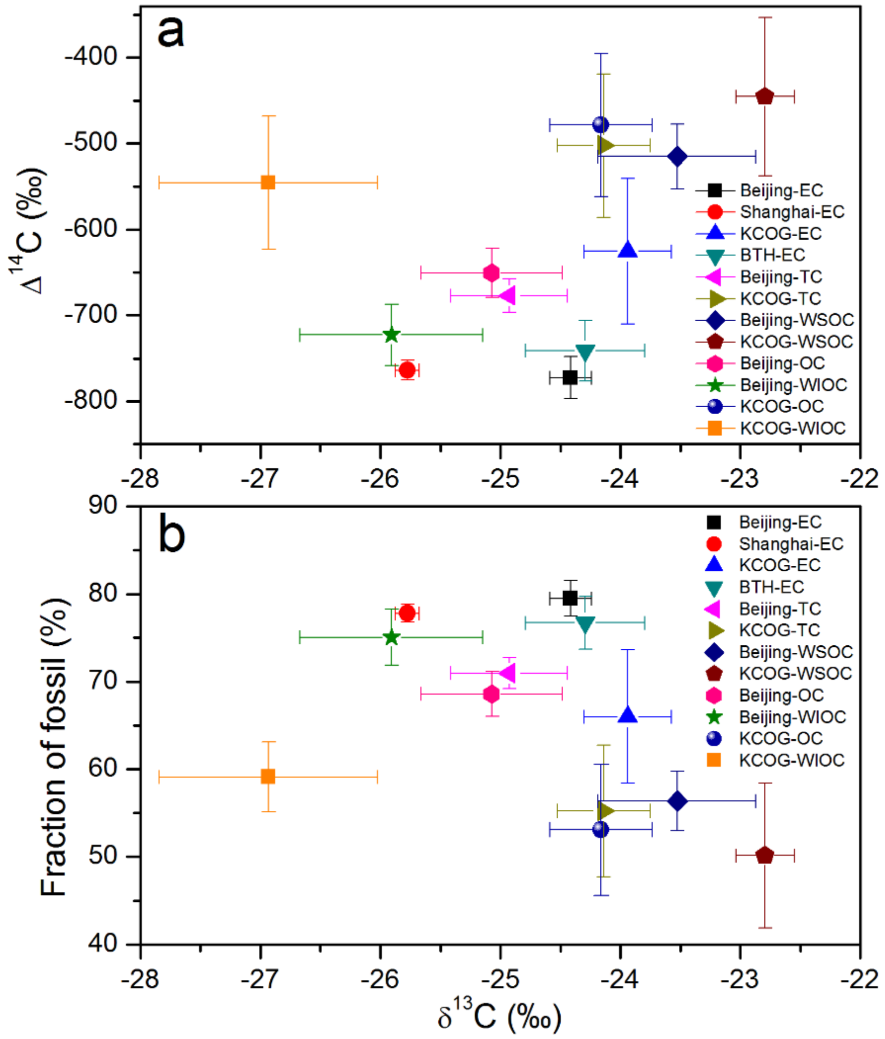

Figure S21. Two-dimensional dual carbon isotope $\delta^{13}C$ versus $\Delta^{14}C$ presentation of carbon aerosol fractions (EC, OC, TC, WIOC & WSOC). (a) $\delta^{13}C$ (mean ± standard deviation) versus $\Delta^{14}C$ (mean ± standard deviation), **(b)** $\delta^{13}C$ (mean ± standard deviation) versus f_{fossil} (mean ± standard deviation,), f_{fossil} was calculated using the $\Delta^{14}C$ signature with a biomass and fossil endmembers of 112‰ and -1000‰, respectively[5]. Symbols indicate mean of all measured samples and standard deviation as error bars.

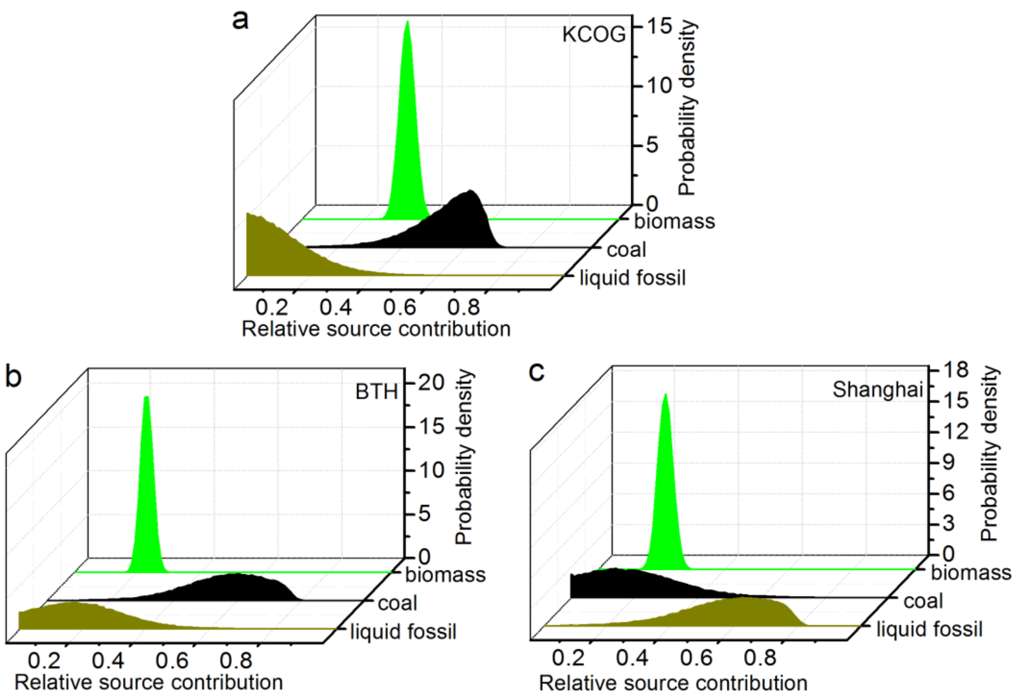

Figure S22. Posterior Probability density functions (PDFs) of relative source contribution of three major sources (liquid fossil, coal, & biomass) for BC aerosols at KCOG, BTH (Wuqing, Tianjin), and Shanghai.

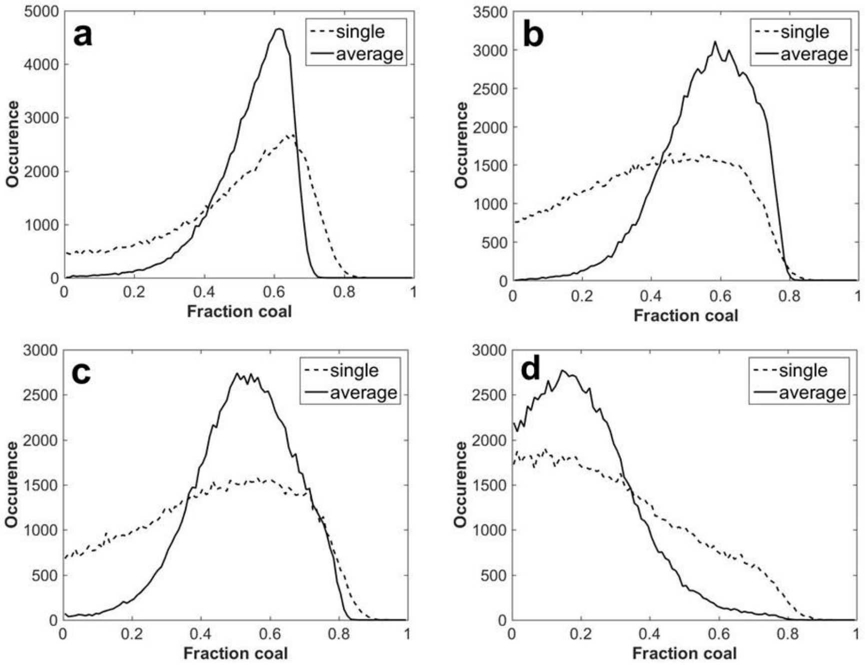

Fig. S23. Occurrences of relative source contribution of coal for BC aerosols at KCOG (panel a), BTH (panel b), Beijing (panel c), and Shanghai (panel d). Dashed line and solid line denote the probability density functions (PDFs) for fraction coal for one data point and the average, respectively.

Table S1. Sampling times, concentrations of EC, OC, TC, WSOC, and $PM_{2.5}$ and, ratios of OC/EC, WSOC/OC, and WSOC/TC for the ambient $PM_{2.5}$ samples in Beijing, BTH, Shanghai, YRD, and KCOG in January 2014 campaign (timing indicated by UTC).

Site-sample ID	Start time (day, time)	End time (day, time)	EC	OC	TC	WSOC	$PM_{2.5}$	OC/EC	WSOC/OC	WSOC/TC
			($\mu g\ m^{-3}$)							
Beijing-0109	09, 01:00	10, 00:30	3.6	17.2	20.8	4.3	-	4.8	0.25	0.21
Beijing-0111	11, 02:45	12, 00:45	7.3	23.3	30.6	7.8	-	3.2	0.34	0.26
Beijing-0112	12, 01:00	13, 00:30	7.0	18.0	25.0	5.8	-	2.6	0.32	0.23
Beijing-0113	13, 01:15	14, 00:45	9.3	32.3	41.6	10.2	-	3.5	0.32	0.25
Beijing-0114	14, 01:00	15, 00:30	4.4	28.1	32.5	8.7	-	6.3	0.31	0.27
Beijing-0115	15, 01:00	16, 00:30	16.9	60.5	77.4	23.8	-	3.6	0.39	0.31
Beijing-0117	17, 01:00	18, 00:30	6.1	23.1	29.2	9.8	-	3.8	0.42	0.34
Beijing-0121	21, 01:00	22, 00:30	5.6	24.9	30.5	8.2	-	4.4	0.33	0.27
BTH-0111	11, 11:45	12, 11:35	3.1	28.2	31.3	8.5	117	9.1	0.30	0.27
BTH-0112	12, 11:45	13, 11:35	11.7	36.2	47.9	12.0	155	3.1	0.33	0.25
BTH-0113	13, 11:45	14, 11:35	8.3	43.4	51.7	12.8	184	5.2	0.30	0.25
BTH-0114	14, 11:40	15, 10:47	11.9	93.3	105	25.9	353	7.9	0.28	0.25
BTH-0115	15, 11:38	16, 11:28	7.4	60.4	67.8	13.3	308	7.8	0.23	0.20
BTH-0116	16, 11:35	17, 11:25	2.4	28.1	30.5	9.9	129	11.1	0.37	0.33
BTH-0117	17, 11:35	18, 11:25	8.0	55.5	63.5	20.4	221	6.9	0.37	0.32
Shanghai-0109	09, 01:21	10, 01:12	1.5	4.8	6.3	2.1	-	3.3	0.43	0.33
Shanghai-0110	10, 01:23	11, 01:47	1.3	6.3	7.6	3.0	-	5.0	0.47	0.39
Shanghai-0111	11, 01:57	12, 01:04	0.9	3.6	4.5	1.9	-	3.9	0.52	0.41
Shanghai-0112	12, 01:16	13, 01:13	1.4	8.9	10.3	5.1	-	6.6	0.57	0.49
Shanghai-0113	13, 01:25	14, 01:24	1.2	3.0	4.2	1.2	-	2.5	0.40	0.28

Shanghai-0114	14, 01:35	15, 01:20	0.9	3.2	4.1	1.3	-	3.5	0.40	0.31
Shanghai-0115	15, 01:30	16, 01:36	1.9	7.5	9.4	3.5	-	4.0	0.47	0.38
Shanghai-0116	16, 01:45	17, 01:15	2.5	10.3	12.8	3.9	-	4.1	0.38	0.30
Shanghai-0117	17, 01:30	18, 02:30	3.3	12.9	16.2	5.9	-	3.9	0.46	0.37
YRD-0103	03, 10:45	04, 10:30	4.9	34.4	39.3	11.2	188	7.0	0.33	0.28
YRD-0104	04, 10:45	05, 10:35	5.7	35.6	41.3	11.6	189	6.2	0.33	0.28
YRD-0105	05, 10:45	06, 10:35	2.7	12.1	14.7	4.7	75	4.5	0.39	0.32
YRD-0106	06, 10:45	07, 10:35	2.8	11.4	14.2	3.6	45	4.1	0.32	0.25
YRD-0107	07, 10:45	08, 10:35	2.4	14.2	16.6	4.2	61	5.6	0.32	0.27
YRD-0108	08, 10:45	09, 10:35	1.2	6.8	8.0	3.0	40	5.5	0.44	0.37
YRD-0109	09, 10:40	10, 10:29	2.3	12.8	15.1	4.7	74	5.5	0.36	0.31
KCOG-0117	17, 00:43	19, 01:29	1.2	7.2	8.4	4.8	-	6.1	0.66	0.57
KCOG-0119	19, 01:50	20, 00:35	0.7	4.4	5.0	2.9	-	6.7	0.66	0.57
KCOG-0120	20, 01:35	22, 01:58	2.5	9.7	12.2	6.8	-	3.9	0.70	0.56
KCOG-0122	22, 02:05	23, 01:14	0.3	1.3	1.6	1.0	-	4.2	0.73	0.59
KCOG-0123	23, 01:20	24, 01:30	0.3	1.4	1.7	0.9	-	4.2	0.66	0.53
KCOG-0124	24, 01:41	25, 00:49	0.2	0.6	0.8	0.3	-	2.9	0.58	0.43
KCOG-0125	25, 01:00	26, 01:26	0.7	3.3	4.0	2.2	-	5.1	0.65	0.54
KCOG-0126	26, 01:33	27, 00:34	0.3	1.5	1.8	1.0	-	5.5	0.64	0.54
KCOG-0127	27, 00:40	28, 00:35	0.2	1.0	1.2	0.7	-	5.6	0.71	0.60
KCOG-0128	28, 00:42	29, 00:51	0.6	2.6	3.2	1.6	-	4.7	0.63	0.52

Table S2. Isotope signatures of EC, OC, TC, WIOC, and WSOC and the corresponding fraction fossil (f_{fossil}) from the daily ambient $PM_{2.5}$ samples in January 2014 E. Asia campaign. The fraction biomass ($f_{biomass}$ = 1- f_{fossil}) and the uncertainty ranges ($f_{biomass}$ /f_{fossil} ≤5%) were estimated by Markov Chain Monte Carlo (MCMC) approach.

Site-sample ID	EC			OC			WSOC			TC			WIOC		
	$\Delta^{14}C$ (-‰)	f_{fossil} (%)	$\delta^{13}C$ (-‰)	$\Delta^{14}C$ (-‰)	f_{fossil} (%)	$\delta^{13}C$ (-‰)	$\Delta^{14}C$ (-‰)	f_{fossil} (%)	$\delta^{13}C$ (-‰)	$\Delta^{14}C$ (-‰)	f_{fossil} (%)	$\delta^{13}C$ (-‰)	$\Delta^{14}C$ (-‰)	f_{fossil} (%)	$\delta^{13}C$ (-‰)
Beijing-0111	780.17	80±1	24.36	622.24	66±1	25.11	546.13	59±1	24.92	659.85	69±1	24.93	660.71	69±1	25.20
Beijing-0112	756.00	78±1	24.28	647.00	68±1	24.72	461.83	51±1	23.16	677.33	71±1	24.60	735.87	76±1	25.47
Beijing-0113	748.57	77±1	24.59	667.83	70±1	26.30	518.67	57±1	23.61	685.85	72±1	25.92	736.85	76±1	27.55
Beijing-0114	774.98	80±1	24.65	659.00	69±1	25.09	491.43	54±1	23.39	674.85	71±1	25.03	733.97	76±1	25.85
Beijing-0115	770.87	79±1	24.45	696.38	73±1	24.65	576.68	62±1	23.15	712.68	74±1	24.61	773.98	80±1	25.63
Beijing-0117	804.08	82±1	24.15	610.68	65±1	24.55	495.76	55±1	22.93	650.90	69±1	24.47	695.00	72±1	25.75
Shanghai-0114	766.99	79±1	25.92												
Shanghai-0115	776.19	80±1	25.65												
Shanghai-0116	745.04	77±1	25.80												
Shanghai-0117	767.39	79±1	25.73												
BTH-0112	721.92	75±1	24.58												
BTH-0113	765.46	79±1	24.59												
BTH-0114	781.27	80±1	24.62												
BTH-0115	712.62	74±1	24.35												
BTH-0116	687.76	72±1	23.20												
BTH-0117	777.17	80±1	24.42												
KCOG-0117	685.61	72±1	23.32	575.51	62±1	24.00	554.04	60±1	22.96	590.96	63±1	23.91	617.95	66±1	26.07
KCOG-0119	612.63	65±1	24.17	454.78	51±1	24.88	440.10	50±1	23.08	475.27	53±1	24.79	483.14	53±1	28.36
KCOG-0120	710.75	74±1	24.23	527.81	58±1	24.00	485.44	54±1	22.67	564.93	61±1	24.04	627.08	66±1	27.09
KCOG-0125	492.76	54±1	24.04	355.37	42±1	23.76	302.45	37±1	22.46	377.96	44±1	23.81	454.62	51±1	26.21

Table S3. Source signatures (endmembers) of radiocarbon $\Delta^{14}C$ and stable carbon $\delta^{13}C$ used in the mass-balance source apportionment calculations (mean ± standard deviation). According to Andersson *et al*. (2015)[7].

	Biomass (C3 plants)	Liquid fossil	Coal
$\Delta^{14}C$ (‰)	112 ± 60	-1000	-1000
$\delta^{13}C$ (‰)	-26.7 ± 1.8	-25.5 ± 1.3	-23.38 ± 1.3

Table S4. Results from the Dual-Carbon ($\Delta^{14}C$ and $\delta^{13}C$) Isotope-Based Bayesian MCMC Source Apportionment Calculations of EC, shown with mean and standard deviation.

	fraction biomass	fraction coal	fraction liquid fossil	fraction biomass	fraction coal	fraction liquid fossil
	mean	mean	mean	s. d.	s. d.	s. d.
KCOG	0.33	0.54	0.13	0.02	0.11	0.11
BTH	0.23	0.56	0.21	0.02	0.13	0.13
Beijing	0.20	0.53	0.27	0.02	0.14	0.14
Shanghai	0.21	0.21	0.57	0.02	0.14	0.15

Table S5. Sampling instrumentation (used for current study) at the Yellow Sea receptor site of Korea Climate Observatory at Gosan (KCOG, Jeju Island, South Korea), regional sites at Wuqing of Beijing-Tianjin-Hebei (BTH) and Haining of Yangtze River Delta (YRD), and the urban sites of Beijing and Shanghai.

Site	aerosol	Sampler or monitor	Manufacturer	Model
BTH (Wuqing)	$PM_{2.5}$	High volume, quartz filter	Tianhong Instrument Co., China	TH-150C III
YRD (Haining)	$PM_{2.5}$	High volume, quartz filter	Tianhong Instrument Co., China	TH-150C III
KCOG (Gosan)	$PM_{2.5}$	High volume, quartz filter	Digitel A.G., Switzerland	DH-77
	PM_{10}	PM_{10} mass monitor		
	$PM_{10,1}$	CLAP[a]	NOAA, CO, U.S.	
	$PM_{10,1}$	Nephelometer[b]	TSI Co., U.S.	3563
(Bongseong)	$PM_{2.5}$	$PM_{2.5}$ mass monitor		
	$PM_{2.5}$	Semi-Online OCEC analyzer	Sunset Laboratory, OR, U.S.	
Beijing	$PM_{2.5}$	High volume, quartz filter	Thermo Fisher Scientific Co., U.S.	VFC-$PM_{2.5}$
Shanghai	$PM_{2.5}$	High volume, quartz filter	Thermo Fisher Scientific Co., U.S.	VFC-$PM_{2.5}$

[a]Light absorption coefficient (Mm^{-1}) measured by Continuous Light Absorption Photometer (CLAP). Wavelength: 467, 528, 652 nm, particle cut-off size: 10 µm, 1 µm.

[b]Aerosol Scattering Coefficient (Mm^{-1}) measured by Nephelometer (TSI model 3563). Wavelength: 450, 550, 700 nm, particle cut-off size: 10 µm, 1 µm.

Table S6. OC, EC, and TC concentrations (μg cm^{-2}) of $PM_{2.5}$ filter samples from Shanghai, measured by a thermal-optical transmission (TOT) analyzer (Sunset Laboratory Inc., Tigard, OR, USA) using the National Institute for Occupational Safety and Health (NIOSH) 5040 method.

ID	OC	EC	TC	OC	EC	TC
	Acid fumigation (μg cm^{-2})			Non-acid (μg cm^{-2})		
0109	20.9	5.6	26.5	19.7	6.1	25.8
0110	27.0	5.4	32.4	26.6	5.3	31.9
0111	14.9	3.2	18.1	14.5	3.7	18.1
0112	38.0	6.6	44.5	36.6	6.8	43.4
0113	12.9	5.1	18.0	12.3	5.0	17.3
0114	13.3	3.4	16.7	13.1	3.5	16.6
0115	31.7	5.8	37.5	31.0	5.8	36.8
0116	42.9	9.3	52.2	41.4	10.2	51.6
0117	57.9	12.8	70.7	56.4	13.1	69.5

Text S1. Descriptions of two regional sampling sites

Wuqing district (near the rural place) is at Tianjin City of China, a representative atmosphere of Beijing-Tianjin-Hebei (BTH; is a part of NCP). The sampling inlet is at above 13-meters height, with no obvious emission sources around except two major roads, distance to nearby streets: SW75 meter, NE350 meter.

Haining is in Zhejiang province of China is a regional receptor site of YRD. The sampling inlet is at above 25-meters height, with no obvious emission sources around, distance to nearby streets: 300 meters.

Text S2. Back trajectories analysis

Five-day back trajectories (BTs) at heights 500m and 100m (a.g.l.) for KCOG (Gosan, Jeju Island, South Korea) were calculated using the NOAA HYbrid Single Particle Lagrangian Integrated Trajectory (HYSPLIT)[4-6] model (Draxler and Rolph, 2003)[4], with 6-hourly archived meteorological data provided from the US National Centers for Environmental Prediction (NCEP) Global Data Assimilation System (GDAS).

Acknowledgment

The authors gratefully acknowledge the NOAA Air Resources Laboratory (ARL) for the provision of the HYSPLIT transport and dispersion model and/or READY website (http://www.ready.noaa.gov) used in this publication.

Supplementary References

1. Kim, S.-W. *et al*. Aerosol optical, chemical, and physical properties at Gosan, Korea during Asian dust and pollution episodes in 2001. *Atmos. Environ*. **39,** 39–50 (2005).
2. Kirillova, E. N., Andersson, A., Han, J., Lee, M. & Gustafsson, Ö. Sources and light absorption of water-soluble organic carbon aerosols in the outflow from northern China. *Atmos. Chem. Phys*. **14,** 1413–1422, doi: 10.5194/acp-14-1413-2014 (2014).
3. Huebert, B. J. *et al*. An overview of ACE-Asia: Strategies for quantifying the relationships between Asian aerosols and their climatic impacts. *J. Geophys. Res*. **108,** 8633 (2003).
4. Draxler, R. R., Rolph, G. D., 2003. HYSPLIT (HYbrid Single-particle Lagrangian Integrated Trajectory). Model Access via NOAA ARL READY. NOAA Air Resources Laboratory, Silver Spring, MD. Website: http://www.arl.noaa.gov/ready/hysplit4.html.
5. Stein, A.F., Draxler, R.R, Rolph, G.D., Stunder, B.J.B., Cohen, M.D., & Ngan, F. NOAA's HYSPLIT atmospheric transport and dispersion modeling system, *Bull. Amer. Meteor. Soc.*, **96**, 2059-2077 (2015).
6. Rolph, G., Stein, A., & Stunder, B.,. Real-time Environmental Applications and Display sYstem: READY. *Environmental Modelling & Software*, **95**, 210-228 (2017). (http://www.sciencedirect.com/science/article/pii/S1364815217302360)
7. Andersson, A. *et al*. Regionally-varying combustion sources of the January 2013 severe haze events over eastern China. *Environ. Sci. Technol*. **49,** 2038–4496, doi: 10.1021/es50385e (2015).
